# A Mobile Phone Intervention to Improve Obesity-Related Health Behaviors of Adolescents Across Europe: Iterative Co-Design and Feasibility Study

**DOI:** 10.2196/14118

**Published:** 2020-03-02

**Authors:** Anne Martin, Maurizio Caon, Fulvio Adorni, Giuseppe Andreoni, Antonio Ascolese, Sarah Atkinson, Kim Bul, Carme Carrion, Conxa Castell, Valentina Ciociola, Laura Condon, Mireia Espallargues, Janet Hanley, Nithiya Jesuthasan, Claudio L Lafortuna, Alexandra Lang, Federica Prinelli, Elisa Puidomenech Puig, Sarah A Tabozzi, Brian McKinstry

**Affiliations:** 1 UK Medical Research Council / Chief Scientist Office Social and Public Health Sciences Unit University of Glasgow Glasgow United Kingdom; 2 Haute Ecole de Gestion and Haute Ecole d'Ingénierie et d'Architecture University of Applied Sciences and Arts of Western Switzerland Fribourg Switzerland; 3 Institute of Biomedical Technologies National Research Council Milan Italy; 4 Design Department Politecnico di Milano Milan Italy; 5 Imaginary srl Milan Italy; 6 Human Factors Research Group University of Nottingham Nottingham United Kingdom; 7 Centre for Innovative Research Across the Life Course Coventry University Coventry United Kingdom; 8 eHealth Lab Research eGroup Universitat Oberta de Catalunya Barcelona Spain; 9 Public Health Agency of Catalonia Barcelona Spain; 10 Division of Primary Care University of Nottingham Nottingham United Kingdom; 11 Catalan Agency for Health Information, Assessment and Quality Barcelona Spain; 12 Health Services Research on Chronic Patients Network Barcelona Spain; 13 School of Health and Social Care Edinburgh Napier University Edinburgh United Kingdom; 14 Institute of Molecular Bioimaging and Physiology National Research Council Milan Italy; 15 Usher Institute for Population Health Sciences University of Edinburgh Edinburgh United Kingdom

**Keywords:** health behavior, obesity, co-design, mHealth, mobile app, mobile phone, adolescents, youth, focus groups

## Abstract

**Background:**

Promotion of physical activity, healthy eating, adequate sleep, and reduced sedentary behavior in adolescents is a major priority globally given the current increase in population health challenges of noncommunicable diseases and risk factors such as obesity. Adolescents are highly engaged with mobile technology, but the challenge is to engage them with mobile health (mHealth) technology. Recent innovations in mobile technology provide opportunities to promote a healthy lifestyle in adolescents. An increasingly utilized approach to facilitate increased engagement with mHealth technology is to involve potential users in the creation of the technology.

**Objective:**

This study aimed to describe the process of and findings from co-designing and prototyping components of the PEGASO Fit for Future (F4F) mHealth intervention for adolescents from different cultural backgrounds.

**Methods:**

A total of 74 adolescents aged 13 to 16 years from Spain, Italy, and the United Kingdom participated in the co-design of the PEGASO F4F technology. In 3 iterative cycles over 12 months, participants were involved in the co-design, refinement, and feasibility testing of a system consisting of diverse mobile apps with a variety of functions and facilities to encourage healthy weight–promoting behaviors. In the first iteration, participants attended a single workshop session and were presented with mock-ups or early-version prototypes of different apps for user requirements assessment and review. During the second iteration, prototypes of all apps were tested by participants for 1 week at home or school. In the third iteration, further developed prototypes were tested for 2 weeks. Participants’ user experience feedback and development ideas were collected through focus groups and completion of questionnaires.

**Results:**

For the PEGASO F4F technology to be motivating and engaging, participants suggested that it should (1) allow personalization of the interface, (2) have age-appropriate and easy-to-understand language (of icons, labels, instructions, and notifications), (3) provide easily accessible tutorials on how to use the app or navigate through a game, (4) present a clear purpose and end goal, (5) have an appealing and self-explanatory reward system, (6) offer variation in gamified activities within apps and the serious game, and (7) allow to seek peer support and connect with peers for competitive activities within the technology.

**Conclusions:**

Incorporating adolescents’ preferences, the PEGASO F4F technology combines the functions of a self-monitoring, entertainment, advisory, and social support tool. This was the first study demonstrating that it is possible to develop a complex mobile phone-based technological system applying the principles of co-design to mHealth technology with adolescents across 3 countries. The findings from this study informed the development of an mHealth system for healthy weight promotion to be tested in a controlled multinational pilot trial.

## Introduction

### Background

Innovations in mobile technology for health (mobile health [mHealth]) provide a novel opportunity for combating child and youth overweight and obesity by promoting behavior change for increased physical activity, reduced sitting time, and healthy eating habits [1]. Recent developments of mobile technology provide personalized, continuous, and context-aware feedback on behavior, which is suggested to be a critical component for health behavior change and potentially increases adherence [2-4]. Integrating game design elements in existing intervention approaches (so-called gamification) and using educational games (ie, serious games) to improve health-related behavioral outcomes appear to be effective in young people [5,6]. However, in the context of promoting a healthy lifestyle among young people, more research efforts need to be directed to successfully promoting adequate engagement with these apps as high levels of disengagement with mHealth technology have been reported [7]. This long-term disengagement will result in nonadoption and eventual abandonment of the technology by intended users [8]. Although young people are becoming more interested in using commercial mobile apps for their health [9], engagement with evidence-based mHealth interventions is low. For example, adolescents participating in a 12-week mobile phone-assisted healthy weight intervention monitored their meals on only 16.6% of available days and tracked at least 30 min of physical activity on only 4.6% of available days [10]. In an 11-week randomized controlled trial for testing a mindfulness app for improving weight-related behaviors, only 14% (5/34) of young adults accessed the content regularly, 61% (21/34) used the app periodically and 23% (8/34) did not engage with the app [11]. This literature exemplifies the challenges and complexity of developing and implementing a successful mHealth intervention. The need and potential benefit of the intervention may be clear, but the initial uptake and long-term use will be contingent on the technology’s ability to eliminate barriers to engagement via integrating and adapting to real-world complexity [8].

One way of tackling disengagement is the user-centered design (UCD) approach, which puts the user, with their needs, desires, and limitations at the center of the design process. The value of this approach is now well recognized, and as such, to improve engagement and uptake of mHealth by young people, they are increasingly being involved in the creation of these technologies. Co-design refers to the collective “creativity of designers and people not trained in design working together in the design development process” [12]. Co-design is an approach that is a subdiscipline of participatory design and can be integrated into a UCD process, ensuring that end users are provided with opportunities to express what they want from a product or service. This can be achieved using qualitative and design methodologies that are tailored to the specific user group in question. Co-design provides designers with the right tools to elicit reliable user requirements and subsequently develop systems that better meet user needs and improve user experience [13]. The importance of implementing this approach and involving multiple stakeholders in the development of mHealth interventions has been demonstrated in the literature supporting mHealth for nutrition management and in its specific application in co-design with young people [14-16].

### Objectives

Recognizing the potential of mHealth as an efficient tool for health behavior change in young people and the need for adequate engagement with the technology, the PEGASO Fit for Future (F4F) project aimed to develop a multidimensional interdisciplinary system placing the target population in the center of development process. The PEGASO F4F system exploited sophisticated game mechanics aiming to motivate behavioral changes toward a healthier lifestyle and prevention of youth obesity. The 3 main features of the PEGASO F4F system were: (1) individual and environmental monitoring, including wearable sensors, mobile phones, and multimedia diaries for the acquisition of physical, physiological, and behavioral attributes of participants; (2) feedback to the user, presenting personalized healthy options for alternative lifestyles; and (3) social connectivity and engagement, encouraging involvement in social network and sharing experiences [17]. The PEGASO F4F system was intended to function on its own and was not part of a wider nondigital healthy weight–promotion initiative or intervention. The entire PEGASO F4F system comprised 8 connected technological components; however, the focus of this paper was to report on the development of 3 system components. The development and content of the remaining components were described elsewhere [18]. All system components were intended to function in conjunction with each other rather than as a standalone application.

The development of the PEGASO F4F system was aligned to the Integrate, Design, Assess, and Share framework for the development of digital technology for health behavior change [19]. The framework consists of 10 phases: (1) empathize with target users, (2) specify target behavior, (3) ground in behavioral theory, (4) ideate implementation strategies, (5) prototype potential products, (6) gather user feedback, (7) build a minimum viable product, (8) pilot test to assess potential efficacy and usability, (9) evaluate efficacy in a randomized controlled trial, and (10) share intervention and findings. Phases 1 to 4 of the PEGASO F4F project were described elsewhere [20,21].

Phases 5 to 7 are the focus of this paper. Therefore, the aim of this study was to describe the process and findings of co-designing and prototyping potential components of the PEGASO F4F mHealth intervention and building a minimum viable product before testing the feasibility and piloting the PEGASO F4F system in a controlled multicenter intervention study (phase 8). Phases 1 to 8 were funded by the European Commission under the Seventh Framework Programme between 2014 and 2017. The research presented in this paper took place between 2015 and 2016.

## Methods

### Study Design

PEGASO F4F was a European multicenter project. Co-design and development of the technology took place in 3 iterative stages in the United Kingdom (England and Scotland), Italy (Lombardia), and Spain (Catalonia). Table 1 summarizes the specific objectives for the PEGASO F4F apps and serious game. The objectives of subsequent iterations were influenced by the findings and the co-design progress of the previous iteration.

**Table 1:** Objectives for each co-design phase.

System component	First iteration	Second iteration	Third iteration
Apps	Assessment of usability and satisfaction, comparison of 2 different prototypes with different interaction layers	Assessment of usability and satisfaction; testing of automatic functionalities, user data entry functionalities	Assessment of usability, intuitiveness, and satisfaction; testing of functionalities
Serious game	Assessment of user needs and acceptance of the visual and graphical content	Assessment of user needs, usability, and acceptance of the game mechanics	Assessment of user needs, usability, and acceptance of the game mechanics; enjoyment, intuitiveness, and playtime

### Participant Recruitment and Study Setting

Male and female adolescents in Spain, Italy, England, and Scotland between the ages of 13 and 16 years were invited through their schools to participate in this study. Convenience sampling was employed for selecting schools. However, schools located in both urban and rural areas and from areas of high and lower deprivation were recruited. Participants were expected to be technologically savvy, that is, with experiences, interest, and ability in using technological devices. This information was based on participants’ self-belief about their interest in and experience with technology. Adolescents whose English language skills did not allow for understanding of the study material were excluded from this study as, for practical reasons, all mock-up and prototype material was available in English language only. In 3 of the 4 study sites, different adolescents were recruited for each iterative stage to ensure the results are generalizable to the general adolescent population. Written study information was provided and written informed consent obtained from the participants directly (Scotland) or from their parents (England, Spain, and Italy). All 3 iterative stages of the technology co-design process were approved by the research ethics committees from the responsible institution in each country: England (Faculty of Engineering Ethics Committee, The University of Nottingham), Scotland (National Health Service South East Scotland Research Ethics Committee 16/SS/0163), Spain (Ethics Committee of Institut Universitari d’Investigació en Atenció Primària Jordi Gol), and Italy (Istituto di Ricovero e Cura a Carattere Scientifico Policlinico of Milan Ethics Committee).

### Concepts of the PEGASO Fit for Future System Components

The development of the PEGASO F4F system was guided by 4 behavioral and theoretical frameworks to raise participants’ awareness about their health behavior and tailor adequate actions for behavior change, namely the Behavior Change Wheel [22], positive psychology [23], self-determination theory [24], and nudging theory [25]. In all, 2 different persuasive mechanisms—gamification and serious gaming—were proposed to allow the participant to engage with the technology while integrating strategies for behavior change [26].

Initial design ideas were derived from the literature and 3 focus groups with 27 young people aged 13 to 15 years [18]. The focus groups explored how young people interact with technology and their preferences for doing so in relation to promoting physical activity and healthy eating, thus informing the prototypes and mock-ups developed for iteration 1 of this study [18].

The entire PEGASO F4F system comprised 8 technological components; however, the focus of this paper was to report on the development of the Companion app, the serious game, and the eDiary app.

### Description of PEGASO Fit for Future System Components

#### Companion App

The objective of the Companion app was to encompass all other PEGASO F4F apps providing a seamless and unique experience to the user. The Companion app was intended to be the app through which the user could engage with all other PEGASO F4F system components. Therefore, users would open one app that in turn contained a suite of functionalities, some of which would be complex apps such as the eDiary and serious game. In addition, the Companion app was intended to make an active lifestyle more attractive, to support users to eat healthier, and to connect users with their peers. To achieve this, notification messages were proposed as a mechanism to facilitate the 3 main functions of the app [27]. The notifications had the potential to be motivational, educational, or acted as reminders to engage with other components of the PEGASO F4F system. For example, notifications reminded the user to enter food items to select a challenge or to nominate a friend for peer support. The challenge component of the Companion app allowed the users to create and accept different types of challenges relating to physical activity and healthy eating. This app offered the opportunity for participants to challenge themselves and other users in a competitive or collaborative way. Previous literature indicated that collaboration and competition can both be beneficial for promoting motivation [28]. The Companion App also included a reward section dedicated to the badges enabling users to unlock new app content and achieve specific goals by using 1 or multiple PEGASO F4F apps and performing the suggested activities. Badges performed multiple functions in congratulating the user on achievements and promoting the social experience of PEGASO F4F through the sharing of badges. This gamification element was intended to generate fun while motivating users to perform specific tasks as part of their interventions.

#### Serious Game

Using a postapocalyptic zombie narrative, the serious game aimed to be the motivational component of PEGASO F4F. It needed to be immersive and engaging for the player, while utilizing the PEGASO F4F system to capture information about lifestyle and encourage maintenance or change of health behaviors. In all, 2 central behavioral mechanisms were envisioned: (1) utilizing behavioral theories of self-determination and nudging, an energy bar indicated the change in energy available for playing the game—the player’s actions in the game reduce energy, whereas achieving real-life behavioral goals such as increased physical activity or improved diet would increase the available energy to play the game, and (2) the game implements *research* mechanics that require the player to apply and develop their nutritional knowledge of various food sources by playing minigames. Minigames were short games embedded within the overall game environment creating opportunities to gain knowledge on food and develop new nutrition-related skills. In the final version of PEGASO F4F, progress within the serious game would be contingent on the physical activity levels of the participant, as measured by the wearable sensors integrated into the system.

#### eDiary App

The objective of the eDiary app was to enable the user to enter information about their usual diet and to provide feedback about healthy eating habits. This app could also provide suggestions to the user on how to improve their dietary habits (eg, increasing or reducing certain food groups and recipes). Informed by literature, researchers with specialist expertise in nutrition, proposed to focus the content of the eDiary app on 6 target behaviors: increased consumption of fruit, increased consumption of vegetables, reduced intake of fast food, reduced intake of sugar-sweetened beverages, healthy snacking, and reduced breakfast skipping. Those behaviors have been selected because of their relevance in obesity prevention and their ability to be modified and measured [29]. The eDiary app allowed the user to select behavioral goals for one of the 6 target behaviors. Users also received automated feedback on the diversity (ie, degree of variation of the diet) and balance (ie, adequacy of food servings relative to recommendations) of the consumed food.

### Co-Design and Technology Development Procedures

The activities, objectives, and duration of each co-design iteration varied depending on the development stage of each PEGASO F4F component. Table 2 summarizes the characteristics of the 3 co-design iterations.

**Table 2:** Components and development stages of the PEGASO Fit for Future Companion app, eDiary app, and serious game.

Stages of system development	First iteration	Second iteration	Third iteration
Mock-up	Companion app and serious game	—^a^	—
Prototype 1.0	eDiary app	Companion app and serious game	—
Prototype 2.0	—	eDiary app	Companion app and serious game
Prototype 3.0	—	—	eDiary app
Integration status	Separate, not integrated system components	Separate, not integrated system components	Partially integrated system components

^a^Progress in development of system components leads to empty cells.

To allow adolescents from a wide range of socioeconomic backgrounds to participate in this study, mobile phone handsets with Android OS version 5.0 (and higher) were provided to participants for the duration of the study.

In the first iteration, participants attended a single workshop session in which they were shown screenshots of the serious game and the Companion app. Participants were presented with the first prototype of the eDiary app, and they were asked to carry out 2 tasks to assess the usability of the app and examine how young people engaged with the early prototype. Task 1 was to modify the entry of a meal, and task 2 was to ask for dietary feedback based on the inserted example meals (example meal plans were provided by the researchers).

During the second iteration, prototypes of both apps and the serious game were trialed on a daily basis for 1 week in the home and school context. The data from the wearable activity monitors were not yet integrated into the serious game because of parallel development work streams. This meant that the serious game was initially tested with in-game progression being dependent on time rather than the physical activity levels of the participant. Future prototype testing (outside the scope of this study) would see the gatekeeping mechanism to in-games progression regarding *energy levels* and accessing new game content being driven by the participants activity levels as measured by the wearable sensors. For this iteration, energy within the game increased slowly over time. This served the purpose of testing if adolescents could be encouraged to engage in short daily game sessions rather than prolonged single-game sessions.

In the third and final stage of the co-design phase, the eDiary and serious game apps were integrated within the Companion app (Table 2). For this iteration, progression in the serious game was based on the player’s physical activity captured by the accelerometer in the mobile phone. Participants of the third co-design iteration tested the PEGASO F4F components for 2 weeks.

### Data Collection and Analysis

Table 3 summarizes the data collection methods for gathering participants’ feedback for each tested PEGASO F4F system component. A mixed method co-design approach was chosen to collect as much feedback in breadth and depth as possible to inform the development of the PEGASO F4F system. In addition, 2 experienced researchers were present during all the workshop and focus group sessions. One researcher was moderating the session and another was allocated to make notes, keep track of the time, and make observations. Although the system components were only available in English, all workshop activities and questionnaires were conducted in the local language.

**Table 3:** Data collection methods used for each PEGASO component at each iteration.

Data collection method	First iteration	Second iteration	Third iteration
Focus groups	eDiary app and serious game	eDiary app, Companion app, and serious game	eDiary app, Companion app, and serious game
System Usability Scale	—^a^	eDiary app and serious game	eDiary app, Companion app, and serious game
Other	App design questionnaire and character design worksheet	Brief Use of App questionnaire and survey on game use	Survey on game use

^a^Not applicable.

#### Design Activities

A character sketchbook with many different proposals for the avatars, icons, and other design elements was provided by developers as a resource for participants to review and develop the Companion and eDiary apps (Multimedia Appendix 1). Vote counting determined the most preferred design.

#### Focus Groups

Focus groups were conducted at all 4 test sites in the local languages and at school and were facilitated by local postdoctoral researchers with a background in behavioral sciences or human-computer interaction (HCI). Facilitators were both male and female in Scotland, Spain, and Italy and all female in England and were not directly involved in the development of the PEGASO F4F software. The focus group topic guides for all 3 iterations can be found in Multimedia Appendix 2 (English-language version only). Focus groups lasted between 60 and 90 min in each iteration, and they were voice or video recorded, transcribed, and thematically analyzed at each study site. Weight of opinion (in terms of number of participants agreeing) was used to make decisions regarding the design, look, and feel or the apps and serious game. Similarities and differences in participants’ views between study sites were identified using thematic analysis, whereby similar feedback across the sites was considered for incorporation in the technology development.

#### Questionnaires

For the development of the Companion app, eDiary app, and serious game, participants were asked to complete 5 questionnaires over the course of the 3 iterative circles (Table 3).

Participants were asked to complete the System Usability Scale (SUS [30]) to determine participants’ subjective rating of the usability of the apps and the serious game. The SUS index was calculated using the standard conversion method [30,31], with scores above 70 indicating acceptable usability, scores between 50 and 70 suggesting marginal usability, and scores below 50 reflecting poor or unacceptable usability [31]. The questionnaire data were entered in to Microsoft Excel and IBM SPSS software (version 21), descriptive summary statistics calculated.

The Brief Use of App Questionnaire (Multimedia Appendix 3) was developed by the research team and included closed and open-ended questions. It consisted of 7 questions relating to the Companion App, of which 3 questions aimed to obtain information on participants’ preferences about the *challenges*, and 4 questions related to the use of the eDiary app.

The Survey on Game Use was also researcher derived and consisted of 4 questions to determine (1) how long participants played the game during a week, (2) when and where they played the game, (3) whether and with whom they talked about the game, and (4) suggestions for the next version of the serious game.

## Results

### Participant Characteristics

A total of 74 adolescents aged between 13 and 16 years participated in the co-design of the PEGASO F4F technology. The number of participants varied between the 4 study sites and between the 3 iterations (Table 4).

**Table 4:** Participant characteristics for each study site and co-design iteration.

Participant characteristics		First iteration	Second iteration	Third iteration
**Participants, n**				
	Spain	8-10^a^	9	10
	England	16^b^	10^b^	10^b^
	Italy	9-10^a^	7-12^c,d^	10^d,e^
	Scotland	11	8-9	5
**Sex (males:females)**				
	Spain	7:3, 2:6	5:4	5:5
	England	6:9	7:3	3:7
	Italy	6:4, 2:7	6:6	4:4
	Scotland	6:5	5:4	3:2

^a^10 participants tested the serious game and 8-9 tested the companion and eDiary apps.

^b^1 participant took part in all 3 iterations, 1 participant took part in the first and second iterations, and 4 participants took part in the second and third iterations.

^c^The number of completed questionnaires varied for different apps.

^d^The same participants took part in the second and third iterations.

^e^10 participants tested the apps.

### Participant Feedback and Design Ideas

#### Companion App

Feedback from the first iteration suggested that the concept of the Companion app was perceived positively and was well accepted by all participants. Participants understood the importance of a central personalized platform to introduce them to the wider PEGASO F4F system. Participants suggested alternative design options for two out of six icons of the Companion app, provided feedback with regards to a PEGASO mascot and a Companion app avatar (Table 5). All participants responded positively to the use of emoticons but suggested the adoption of existing libraries (eg, WhatsApp). The concept of data sharing was an important topic for which participants had clear views. Participants suggested that sharing of the full name and a profile was appropriate at all times. However, data on eating habits should not be shared with peers at any point. As a default, this was agreed, with the addition of modifiable settings that were controlled by the user to share this information but only when and with whom they wanted.

**Figure 1:**
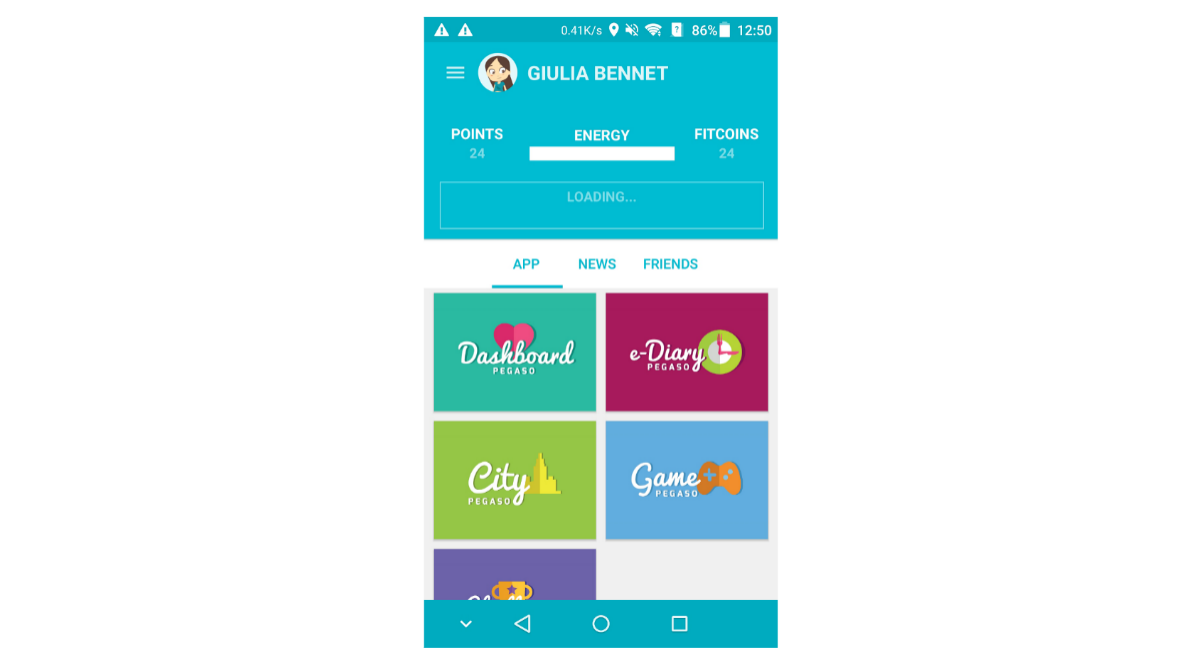
Screenshot of the PEGASO Fit For Future Companion app presented at the third iteration.

Participants who tested the next version of the Companion app in the third iteration of the study (Figure 1), correctly understood the key functions and reported that it was easy to use. They found it visually attractive, helpful, and usable. The average SUS scores indicated marginally acceptable usability of the app in Italy 63.0 (SD 15.1), Scotland 60.0 (SD 17.3), and England 63.3 (SD 36.2) and poor acceptability in Spain 43.7 (SD 4.2). Participants reported that the Companion app was useful as a single app portal for all PEGASO F4F components. However, some of the participants accessed other PEGASO F4F apps bypassing the Companion app. Other participants did not realize that it was possible to collect points and rewards by using the Companion app. Participants of the third iteration liked the idea of being able to set progressive challenges and their own goals, broadening the variety of challenges and sorting them by areas such as *sports* or *eating*. Checking personal progress was considered as motivating for engagement in a healthy lifestyle. They also suggested getting real-life rewards for every challenge met, for example, using social media for setting challenges, where invitations to sport events could be sent and shared.

When using the first prototype of the Companion app in the second iteration, participants experienced difficulties in logging on and in getting the app running. Once it was in operation, participants used the app several times a week and found it easy to use. Participants liked receiving notification messages but expressed concerns about the number and timing of incoming messages (Table 5). For example, adding dietary information to the app while being in class. Participants liked the messages to be short and cheerful in tone, aiming to motivate and provide reminders. Regarding the feedback on health behavior change, most of the participants said that being rewarded with medals that were visible to friends would be seen in a positive way as being motivating to them. The iterative co-design of the visual elements of the interface meant that participants liked the aesthetics of the Companion app; they found the layout was easy to navigate and was easy to read. Most participants found that the amount of information and arrangement on the screens was logical, with clear sequencing of the screens and a predictable ordering of what the next screen advancement would be, suggesting that the early app development complied with the HCI usability principles of visibility of system status and match between system and real world and positive aesthetic experience [32]. Participants liked the idea of being able to set challenges for their friends, compete with each other, and share the results.

**Table 5:** Participants’ co-design output and implemented solution for each PEGASO Fit for Future system component.

Component and participant feedback		Implemented solutions
**Companion app**		
	Not understanding the meaning of the Health Square app icon and Challenge icon; alternative icon design suggested.	The Challenge icon was changed.
	Liked the idea of having a mascot and selected a favorite design.	A Mascot was added based on the design selected by the majority of participants.
	Having a customizable avatar.	Avatars were added based on selected design.
	Not wanting to share data around eating habits.	Sharing of information was limited with control on whether sharing and when.
	Some challenges were difficult, and some were easy; suggested to include incremental challenges.	Incremental challenges implemented.
	Participants suggested to include a leader board as they would be more motivated to engage in health behaviors when seeing their friends’ performance.	Leader board was added.
	Did not like to receive too many notifications or messages.	The number of notifications was fixed to 2/4 messages per day.
	Frustration when receiving notification when it is not possible to respond; suggested to receive notifications in after-school hours.	Notifications timed to be released to later in the day (after school).
	Needing a tutorial or guidance for use of the app.	Tutorial messages implemented.
	Participants did not realize that it was possible to collect points and rewards.	Added a tutorial message and an explanation in the *Points* section.
	Bypassing the companion app to use other PEGASO F4F apps.	Bypassing the Companion app to access other PEGASO F4F apps will not be possible as soon as all remaining PEGASO F4F system services are integrated.
**Serious game**		
	Did not understand the story narrative of the story; suggested to add a tutorial on what the game is about.	A video introduction and tutorial were created.
	The game world is too easy to explore (suggested to provide further dynamism and complexity; specific design ideas for environments or locations provided).	New locations were added. The number of locations increased from 1 to 4.
	Repeating the same minigame was boring.	2 additional minigames were included (scavenging and research).
	No ranks and competitive elements; suggested to include a leader board.	Leader board was implemented.
	Some participants noted they were progressing to higher levels, but they did not notice clear differences between the levels; suggested to add new type of zombies, environments, and abilities (specific ideas provided) with each level.	The possibility to unlock (in different levels) new zombies, new environments, and new abilities were added.
	Suggested to add audio features and sounds.	Music and audio effects were added to increase engagement.
	Feeling that actions in the game had no concrete relationship to the Companion app; suggested to gain coins that can be used in other parts of the PEGASO F4F system.	Fit coins to be used in other sections in the Companion app were introduced.
**eDiary app**		
	It was difficult for adolescents to understand the servings and food included in a group; suggested to include a tutorial.	A help function in form of a question mark icon was added to provide guidance for the food input.
	Preference of symbolic food icon over food images.	Implemented the icon interface instead of the one based on food images.
	Some icons were difficult to be interpreted; alternative images were suggested.	Some food icons (eg, fried food, soft drink, Asian food, and snacks) were redesigned following participants’ suggestions.
	Selected a favorite design for the graphical feedback on diet.	Design preference implemented.
	Not understanding the meaning of “equilibrium.”	“Equilibrium” changed to “balance.”
	Not understanding the meaning of the “diversity” and “balance.”	Explanation of the indexes added.
	Wanting suggestions on how to improve their diet.	Generic recommendations were shown in the News Stream and personalized recommendations were implemented in the eDiary.
	More instructions were needed.	Tutorial cards as instructions and improved help text added.
	Participants proposed to increase the number of food groups and suggested alternative food groups.	Food groups were restructured adding new icons. For example, the food groups *fast* food and *snacks* were represented in more detailed food icons for “Burger,” “Oriental,” “Chicken,” “Pizza,” “Salty,” “Bakery,” “Sweets & Dessert.”
	Preference of receiving advice on which foods should be eaten if a person has food allergies or intolerances.	This was not implemented because of lack of resources.

#### Serious Game

Based on the feedback from the first iteration, participants were positive about the main idea of the game, mainly because of the novelty of the topic and the setting of the game. They liked the idea that they needed to move and to spend energy in real life to gain more energy in the game, a concept which was only tested in the third iteration (see Multimedia Appendix 4). Participants felt that the game was suitable for a mobile phone. On the basis of the mock-up presentation, it became evident that some aesthetic elements should be improved, but the icons and the images were understandable in general. There was a lack of consensus regarding the graphics of the game: some participants preferred the cartoon style of game characters and others the realistic and more sophisticated look of characters. Participants suggested that they could be more engaged with the game if they could interact with other players. They said that they would prefer to customize their avatar (eg, gender, physical appearance, armor, and weapons), to choose their attributes, and to increase skills during the game.

Most of the participants played the serious game between 1 and 3 hours during the weeklong second iteration of this study. Participants played the game in a variety of locations and most of them at home, indicating that the game was usable and acceptable in the daily context of young people’s lives. The suggested features that they wanted to see in the next version of the game included (1) more worlds to explore, (2) more types of zombies and characters, and (3) rewards for healthy real-world actions. Some participants asked for tutorials and help during the game because it was unclear how the game worked and what the aim of the game was. Regarding the game play mechanics, most of the participants felt the daytime activity of finding foods was boring. They suggested adding missions or other game features, such as exploring the inside of the buildings or driving cars. Participants asked for a variety of weapons, more variety of in-game characters, ways to fight zombies, and ways to increase their character abilities. Some participants did not understand how to play the *minigame* and what to do with the recipe cards. The participants felt that they needed more instructions on how to do different aspects of the serious game. The usability of the game using the SUS scale was rated as marginally usable by participants in Spain (mean 67.8, SD 18.2), Italy (mean 62.8, SD 10.8), and Scotland (mean 66.8, SD 17.1). However, participants in England rated the game as having poor usability (mean 20.3, SD 12.0).

Most of the participants of the third iteration of this study liked the concept of the game and said that the controls were easy to use. However, they found that the controls reacted too slowly, and the game was considered repetitive with a lack of progression or a clear end goal. Some participants noted progressing to higher levels in the game, but they did not notice a clear difference between levels. The need of a tutorial within the game was expressed again to understand the aim of the game and *minigames* and how to play them. The written manual provided in response to feedback of the second iteration did not offer adequate assistance to participants of the third iteration. Participants also suggested including different scenarios, more activities, and a clear goal or different *minigoals* that can be unlocked after gaining points. In addition, participants suggested having different settings that could change as the player progressed through the game as a way of reducing repetitiveness. The revised minigames were perceived as visually attractive and usable. However, the participants did not welcome repeating the same *minigame,* and they suggested having additional minigames. Perceptions about the usability of the game using the SUS were mixed across countries. The serious game reached acceptable usability in England (mean 75.0, SD 6.4), marginal usability in Italy (mean 54.3, SD 12.2) and Scotland (mean 57.5, SD 21.1), and poor usability in Spain (mean 45.3, SD 14.1).

#### eDiary App

The idea of the eDiary app was welcomed by females more than males after testing the first prototype of the app in the first iteration of this study. Participants expressed that they would like a tool that is able to collect information about nutritional contents of foods and beverages, to indicate daily dietary balance, and to advice on which foods should be eaten if a person has food allergies or intolerances. The initial food icon designs were well understood by most participants; however, where there was confusion or low satisfaction with the proposed designs, the co-design process enabled participants to offer changes and suggested icons to improve clarity. Participants also expressed their preference of the design of the graphical feedback display. Participants said that they preferred a user interface that was more symbolic rather than displaying photos of food items. Several participants proposed to increase the number of food groups and to reduce the number of foods included in each food group. Participants liked the idea of receiving feedback on how balanced their daily diet was.

During the second iteration, all participants used the eDiary app, and generally, females were more willing to use this app regularly than males. All the participants noted that the food groups were too limited and sometimes did not fit with the information that participants wanted to enter and felt that the food groups should be better explained. Participants noted that entering information on food consumed was difficult at times, leading to frequent mistakes that were difficult to erase; thereby, indicating that the system did not enable users to recognize, diagnose, and recover from errors as is recommended in the HCI literature [32]. Moreover, participants did not like the negative feedback when the food intake was not healthy. They suggested the app should tell them how to improve their eating habits. Mean SUS scores indicated marginal usability across all countries: Spain (mean 58.3, SD 18.6), Italy (mean 70.0, SD 14.6), Scotland (mean 69.1, SD 16.4), and England (mean 59.3, SD 7.6).

**Figure 2:**
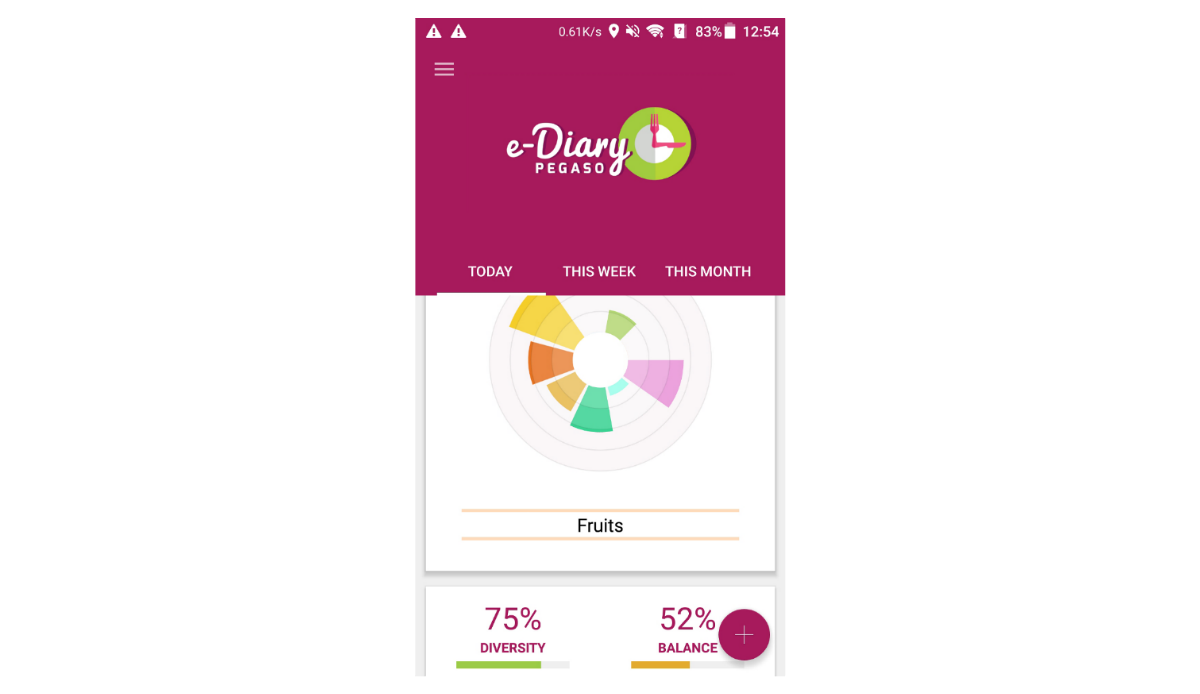
Screenshot of the PEGASO Fit For Future eDiary app presented at the third iteration.

Participants who used the revised prototype version of the eDiary app in the third iteration (Figure 2) found the app easy to understand and aesthetically pleasing. All the participants had used eDiary and agreed that they liked it. This feedback aligned with the improved usability ratings, with average SUS scores of 61.2 (SD 7.0), 75.0 (SD 16.8), 71.5 (SD 13.5), and 76.8 (SD 16.7) in Spain, Italy, Scotland, and England, respectively. Participants liked to get feedback about their food intake and the visualized feedback in form of a pie chart (Figure 2) helped them to increase their perceived awareness on their diet. Participants would have liked to see recommendations for improving eating habits based on the information entered. It seemed to be important to the participants to receive reminders for entering information about consumed food. Some participants mentioned that it would be helpful to be able to enter the exact amounts of food that was consumed to improve accuracy of the feedback. Participants liked the idea of creating their own goals, such as reaching a certain percentage of diversity across food groups, and they were interested in getting feedback on their target goals. They suggested receiving this feedback weekly or every few days. Almost all participants had difficulties understanding the meaning of the feedback around balanced diet and diet diversity and the meaning of the term *equilibrium*; they suggested to change the terminology or to provide an explanation.

### Implications for the PEGASO Fit for Future System Development

Table 5 summarizes the participants’ design ideas for improving and changing the PEGASO F4F system components described in this paper and how the feedback was implemented for each technology. Quotes to support the findings are provided in Multimedia Appendix 5. Some of the design suggestions brought forward by the participants could not be implemented because of technical or practical reasons. For example, customizing the *challenges* function for each target behavior was deemed as too demanding in terms of time and resources for the development of the PEGASO F4F system. The option of exchanging points acquired by engaging with the PEGASO F4F system for rewards in the real world has been explored by the consortium but proved not to be viable at this stage of the intervention development.

For the development of the eDiary app, the SUS scores were used to track the evolution of the usability of the app and to ensure that the interface remained acceptable for participants. Changes made to the interface of the eDiary app in response to testing the app in iteration 2 (Table 5), yielded in improved SUS scores for the eDiary app from iteration 2 to iteration 3. This indicated that the eDiary app gained in usability and functionality. SUS scores for the Companion app were obtained in the third iteration only, and they indicated acceptable usability of the app.

SUS scores for the serious game indicated marginal usability for participants of 3 out of 4 test sites in the second iterations. SUS scores dropped in the third iteration in those 3 countries. Low SUS score for serious games are not uncommon in adolescents because they (1) implicitly compare serious games with commercial games [33] and (2) typically play action games on consoles or personal computers rather than mobile phones [34]. For this reason, the development of the serious game was primarily guided by focus group feedback.

## Discussion

### Principal Findings

This study aimed to describe the process of and findings from co-designing and prototyping components of a mobile phone-based technological system for promoting a healthy diet, physical activity, and reducing sedentary behavior in adolescents. Findings of this study suggested that for the PEGASO F4F technology to be motivating and engaging to adolescents, it should (1) allow personalization of the interface, (2) have age-appropriate and easy-to-understand language (of icons, labels, instructions, and notifications), (3) provide easily accessible tutorials on how to use the app or navigate through a game, (4) present a clear purpose and end goal, (5) have an appealing and self-explanatory reward systems, and (6) offer variation in gamified activities within apps and the serious game. Our findings also stress the importance of the ability of the mHealth technology to seek peer support and connect with peers for increased motivation and engagement with both mobile phone apps and the serious game through collaborative and competitive elements such as leader boards and joint activities. Incorporation of these user requirements led to the development of PEGASO F4F technology that combines 4 functionalities that are rarely united in currently existing mHealth technologies for healthy weight promotion in adolescents: (1) assisting the user in tracking and reviewing their behavior, (2) persuading the user to adopt intended behaviors through a serious game and gamification of apps, (3) advising the user on how to adopt intended behaviors, and (4) providing a platform for the user to interact with peers to encourage adoption of intended health behaviors. Therefore, PEGASO F4F advanced the current state of the art and existing body of literature on mHealth for obesity prevention in young people.

### Comparison With Prior Work

The current literature on the development of mHealth technology for prevention of obesity typically focused its efforts on a single app, game, or behavior (diet or physical activity), thereby ignoring the importance of promoting reduced sedentary behavior, and studies typically involved small numbers of participants in a single country [1,4,35,36]. PEGASO F4F is the first study that applied the principles of UCD of mHealth technology for adolescent healthy weight promotion at scale: co-design and feasibility testing of 3 different system components (2 apps and a serious game) for 3 health behaviors with 74 adolescents in 3 iterative cycles over the course of 12 months in 3 countries.

To date, studies reporting 3 or more cycles of iterative mHealth technology development for healthy weight promotion with adolescents are scarce; thus, the design of function and features of apps and games tend to rely on perceptions of the designers on users’ needs and requirements [37]. The lack of iterative circles of technology design may result in a substantial level of uncertainty in usability, satisfaction, and acceptability of the new technology. With limited resources for research and concerns of producing ineffective interventions, intervention developers are urged to invest in thorough development of interventions involving the intended user populations before piloting the intervention and study design procedures in a controlled experimental study [38]. Taking the time and resources to incorporate adolescents’ ideas, needs, and preference and present them with further developed technology twice, resulted in increased confidence of the PEGASO F4F consortium for producing an engaging mHealth technology for healthy weight promotion in a diverse group of adolescents. This was the case despite limited improvement of the SUS-obtained usability scores for the 3 apps reported in this paper. Although the eDiary app and serious game were integrated with the Companion app, other PEGASO F4F system components (eg, wearable technology and dashboard app) were not fully integrated during the third and final iteration of the co-design study. It is likely that this influenced the user experience and usability when engaging with the apps and serious game. In particular, the wearable technology was an important part for the functionality and user experience of the serious game linking real-world physical activity to the serious game (further explanations go beyond the scope of this paper). However, it is important to note that each co-design iteration involved different young people in most study sites and, as noted above, not all feedback and views could be incorporated. Therefore, the co-design process presented a series of compromises which was tested in the next phase. The next phase of the PEGASO F4F project tested the usability and acceptability of all fully integrated system component.

The approach taken for developing the PEGASO F4F system was in line with most research demonstrating end-user involvement (ie, as informants or co-designers). Therefore, involving adolescents in the development of the PEGASO F4F system as informants during 3 iterative focus group sessions in which they could verbalize their requirements of the system and ideas and feelings about the technology was considered a valid approach. However, it should be noted that results of a meta-analysis suggested that an active co-design role does not always increase serious game effectiveness as users often cannot relate the app or serious game to its learning objectives [28]. To combat this, the iterative stages included multiple ways for adolescents to (1) critically analyze their use and experience of the system, (2) provide feedback, and (3) contribute to the co-design process. By offering multiple methods to participants with differing capabilities and strengths, it increases the opportunity for each stage of technology development to capture valuable and accurate data from a population with wide intra- and intervariability [39]. Previous work on serious games has experienced similar variation in the output and value of end user involvement throughout the design process [40]. A concept introduced by Khaled and Vasalou [41] suggests a finer approach to participant selection in co-design, differentiating contributions in terms of domain expertise and procedural aspects to overcome some of the limitations that hinder current contributions to the design process; however, this approach has not been extensively tested. The authors consider that there is much more to learn about the application of participatory design and co-design methodology in the conceiving, design, development, and implementation of serious games.

Some design ideas could not be implemented for technical and practical reasons; however, this brings the research study in line with real-world development projects for which financial and time constraints are a common challenge. Lean development methods aim to overcome some of those challenges; however, in practice, research and development projects cannot always keep to these more rigid cycles because of their exploratory nature and work in innovative spaces.

### Study Limitations

Focus groups and questionnaires were used for collecting design ideas and feedback on the usability and acceptability of the PEGASO F4F technology, and feedback guided the development of the apps and serious game. Although the use of focus groups and questionnaires reflects a solid approach to ascertain usability, additional methods need to be considered when refining the PEGASO F4F apps and serious game in future research.

First, the use of think aloud methods while adolescents are playing the game and using the apps will give more information about usability issues and the cause of these problems [42]. This will also minimize the chance of missing information because of recall bias, given that adolescents would play the game and verbalize feedback immediately instead of reporting experiences through questionnaires and interviews retrospectively.

Second, collection and interpretation of system usage data can provide valuable information in terms of measuring the level of engagement with mHealth technology [43]. A mixed method approach is recommended during mHealth technology development in which quantitative data (eg, back-end system data) reflect objective usage and qualitative data (eg, semistructured interview) provide more insight into reasons for playing or using, or more importantly, not playing the game or using apps [43]. This would give designers and researchers a better and a more concrete understanding on which elements to adapt, and how to translate adolescents’ feedback into engaging age-appropriate app and game design [5].

Third, to benefit from current efforts in gathering adolescents’ feedback, it is recommended to get a designer involved during feedback sessions. As part of a multidisciplinary interview team, they would have a good understanding of game dynamics and ask appropriate follow-up questions that could be valuable for improved game design and possibly effectiveness. This would be in line with previous research results indicating that gathering feedback from end users should prioritize game dynamics (eg, level) above game aesthetics (eg, storyline and character) as this seems more strongly related to game effectiveness [40]. Involving game and app developers in the focus groups across the 3 countries and 4 intervention sites was considered for this study, but it was deemed not feasible because of constraints of time, proficiencies in local languages (ie, Catalan), and financial resources.

Another limitation of this study was that all PEGASO F4F prototypes were available in English language only. Although none of the adolescents approached to participate in this study (entire year groups) was excluded because of insufficient English language proficiency, participants in Spain and Italy stressed the importance of having apps available in local languages. The translation of the apps into Italian, Spanish, and Catalan was anticipated from the onset of the PEGASO F4F project and was done for testing the technology in the subsequent controlled pilot study. The lack of multilanguage prototypes might have impacted on participants’ engagement with the technology. However, the design workshops and focus groups were conducted in the local language, allowing participants to express their creativity, ideas, and preferences for the development of the technology. Translation and back-translation of the apps and serious game content was not feasible during the technology development process because of the tight project timeline and constraints in the budget.

### Study Implications and Lessons Learned

This study informed the PEGASO F4F system to be tested in a controlled multicenter pilot study. It provided 3 co-designed system components, the Companion app, eDiary. and serious game. In the PEGASO F4F controlled pilot study, adolescents across 4 European study sites tested the integrated system comprising the Companion app (including the Challenge function), eDiary app, Dashboard app, the serious game. and the wearable (smart garment and wrist-worn activity tracker). The individual system components of the pilot study were described in detail in the pilot study protocol [44].

The findings of this study might be of interest to other researchers and technology developers working in the field of mHealth intervention development for an adolescent population. Especially those developing mHealth interventions for promotion of physical activity and healthy eating in the context of obesity prevention or treatment could consider the following lessons learned:

- mHealth technology for adolescents should allow personalization of app interfaces, have instructions that describe the purpose of and navigation through the app, and offer rewards.
- The language used in the instructions and content should be age appropriate, and researchers should test the appropriateness of the language with the potential user. Adolescents particularly dislike repetitive tasks, and so mHealth technology should avoid unnecessary repetitions and offer variations in activities and content.
- For mHealth technology to be engaging, it should enable adolescents to connect with peers for support in adopting behavior change.

### Conclusions

Incorporating adolescents’ preferences, the PEGASO F4F technology combines the functions of a self-monitoring, entertainment, advisory, and social support tool. This study demonstrated that it is possible to employ an iterative co-design approach for development of a complex mHealth technology for adolescent healthy weight promotion in an international context. Participants’ involvement over 3 iterative co-design circles informed the PEGASO F4F system to be tested in a controlled multicenter pilot study.

## Appendix

Multimedia Appendix 1 Companion app and eDiary app co-design worksheet.

Multimedia Appendix 2 Focus group discussion guides.

Multimedia Appendix 3 The brief use of app questionnaire.

Multimedia Appendix 4 Serious game demo video.

Multimedia Appendix 5 Participants' quotes.

## Abbreviations

F4F: Fit for Future
HCI: human-computer interaction
mHealth: mobile health
SUS: System Usability Scale
UCD: user-centered design
