# Supplementary material for: A Mobile Phone Intervention to Improve Obesity-Related Health Behaviors of Adolescents Across Europe: Iterative Co-Design and Feasibility Study
Source: JMIR Mhealth Uhealth. 2020 Mar 2;8(3):e14118. doi: 10.2196/14118 (PMC7076410; doi:10.2196/14118)
Supplement: Multimedia Appendix 1 [file mhealth_v8i3e14118_app1.pdf]

Participant ID:

Date:

PEGASO

App Companion icons, look and feel and navigation

Neosperience

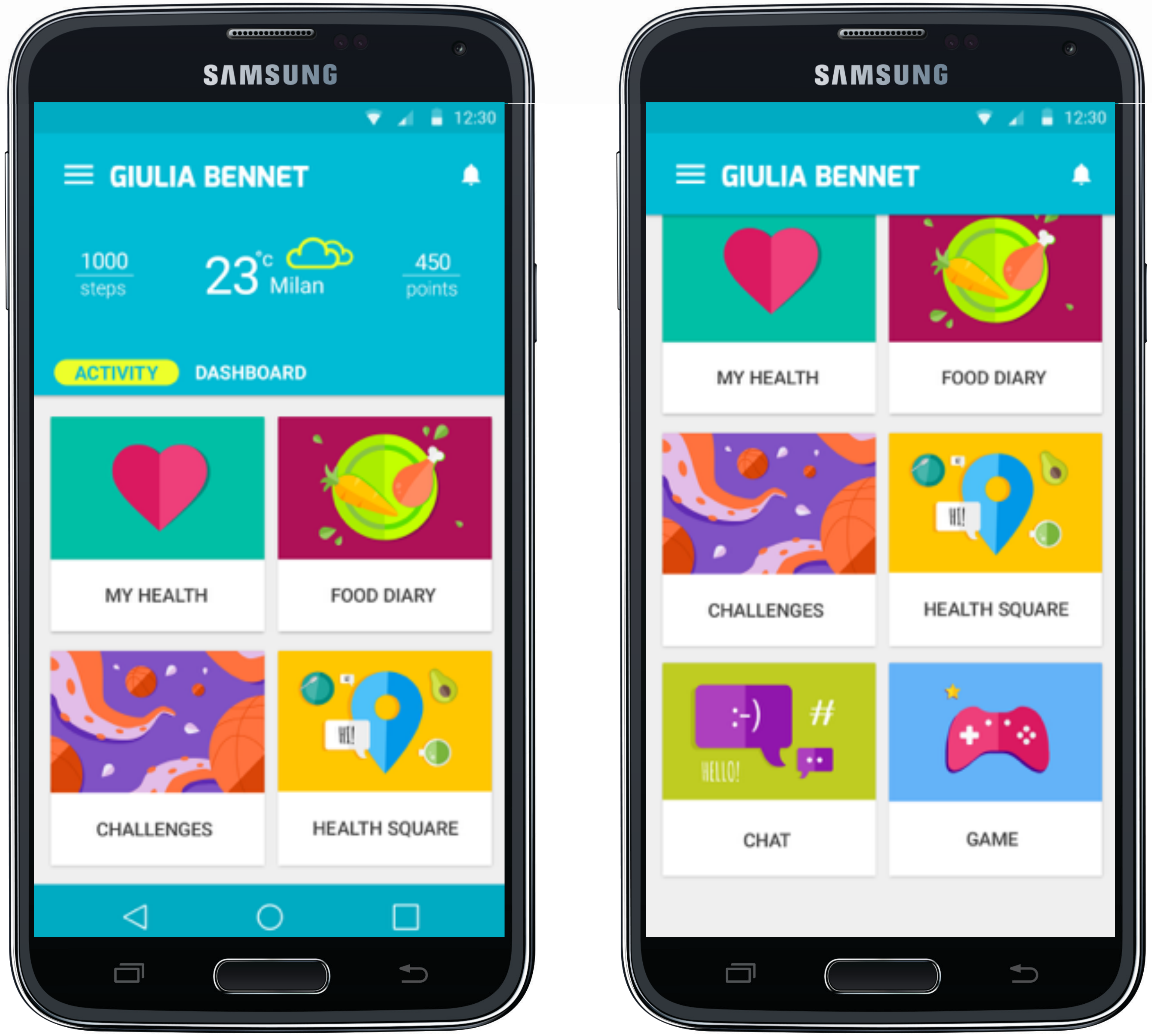

OVERVIEW

The app home screen shows all information about the registered user, and some other useful data: steps already walked, weather forecast, points collected with the challenges.

We ask the participants to test the ease of comprehension of the icons in all sections of the app. Do these icons graphically present the main sections of the app? Do they clarify the different areas?

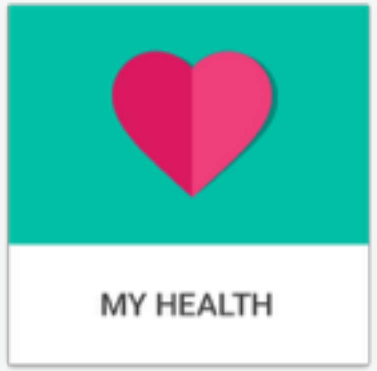

User personal information:

Is the icon appropriate?

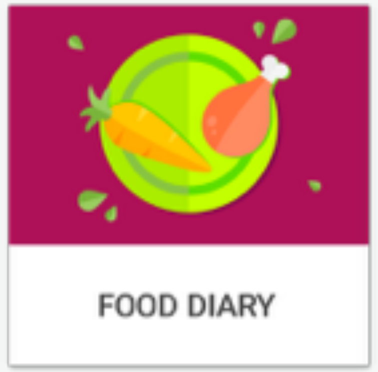

Food diary creation:

Is the icon appropriate?

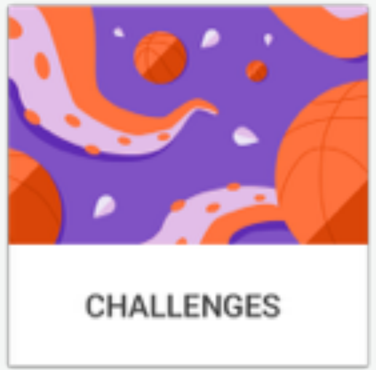

Choose and share challenges with your friends: Is the icon appropriate?

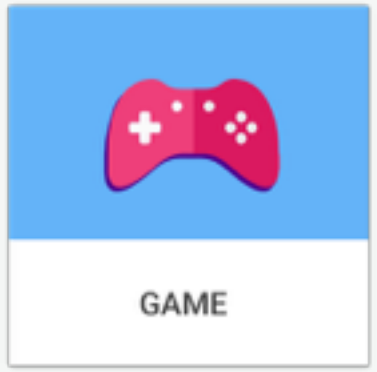

Link to the videogame:

Is the icon appropriate?

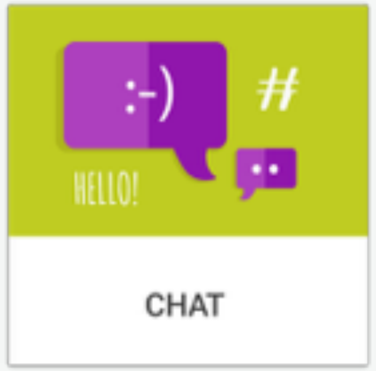

Communication between users:

Is the icon appropriate?

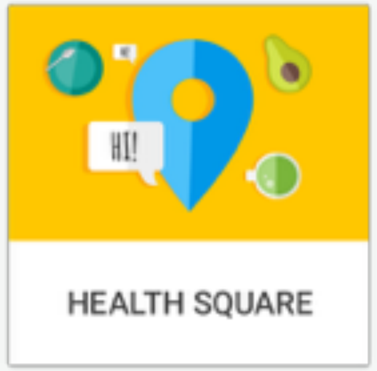

Map to find stores, restaurants and other places: Is the icon appropriate?

We ask the students to test the look and feel of the user dashboard, showing all sections and activities already started.

What do you think about the mix of contents and interactions:

1. there are too many/too few interactions.
2. there are too many/too few contents.
3. there is a balance between interactions and contents.

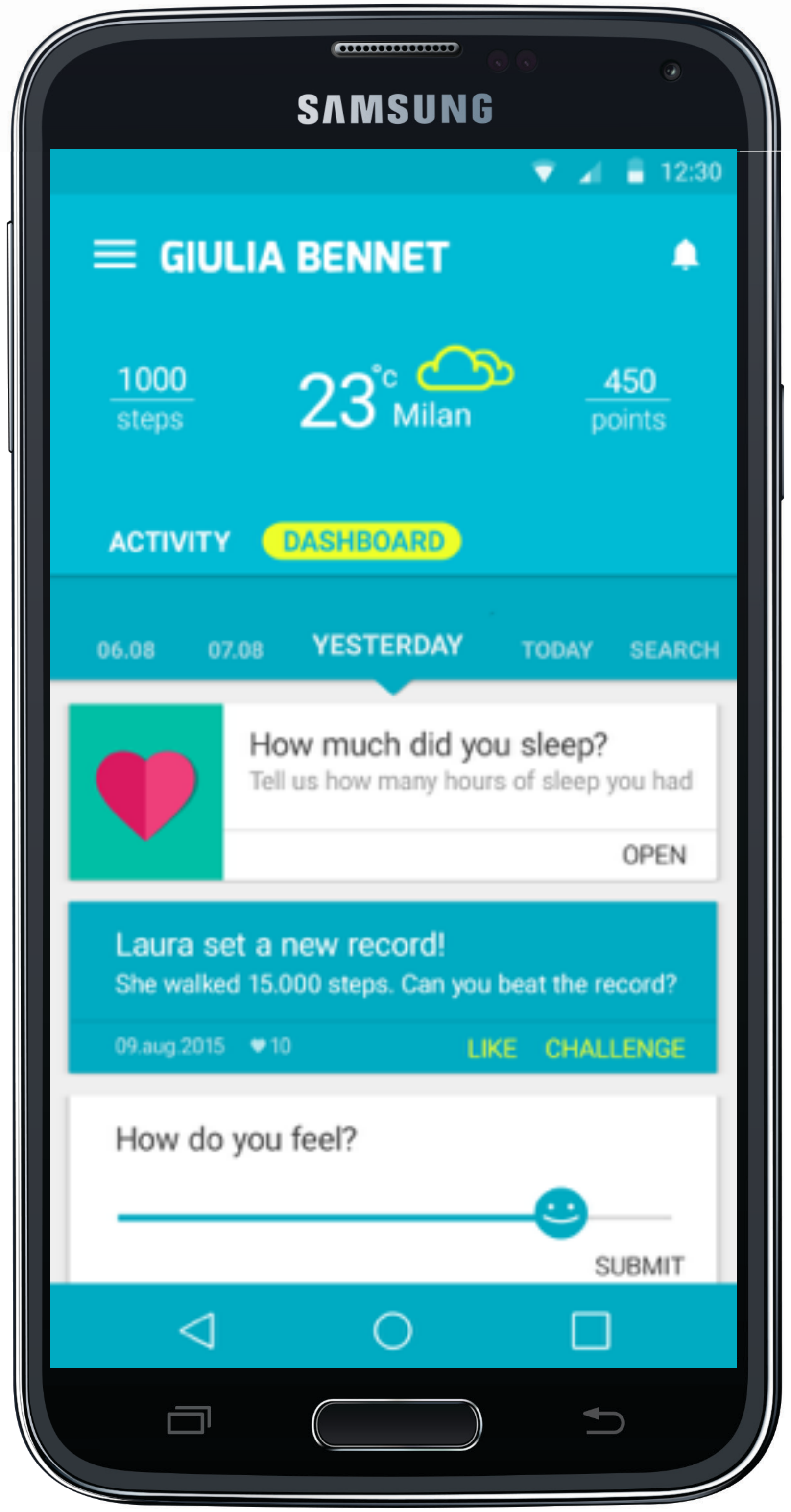

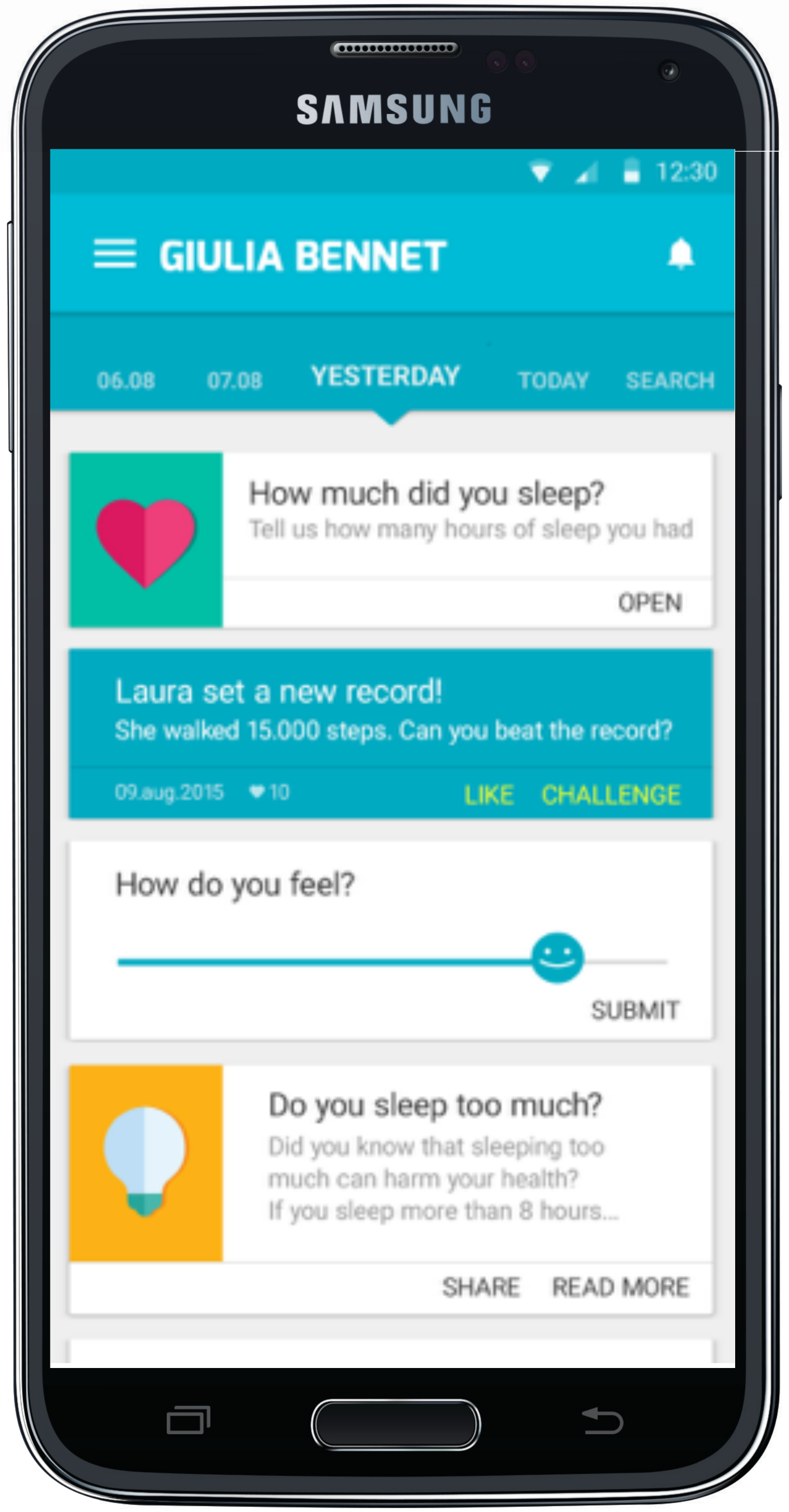

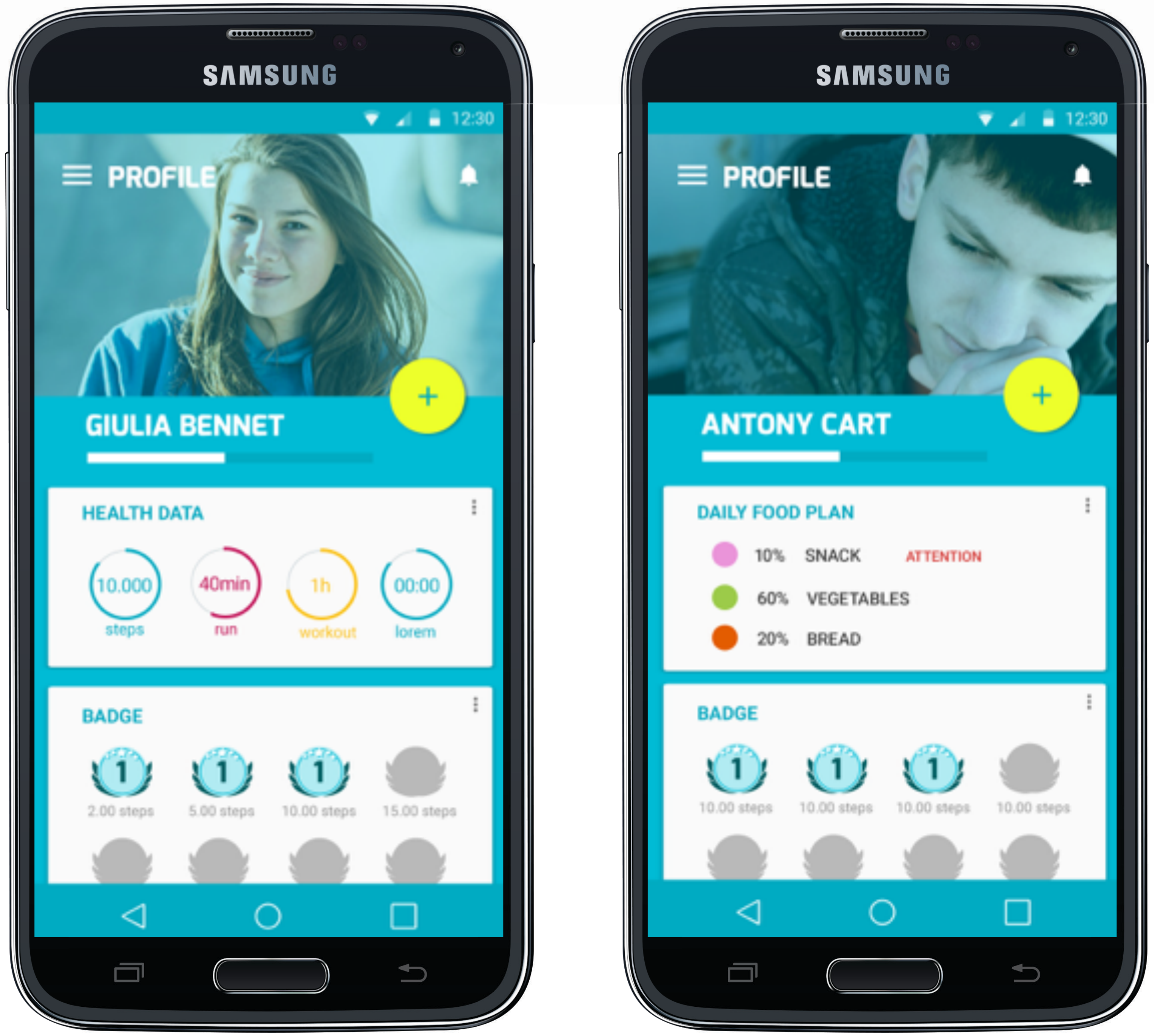

We ask the students to specify what types of information about their profile they'd like to share with the community.

A. Personal information: name, last name, photo

◯ Yes, all ◯ No, none ◯ Part of them

B. Health data: daily steps, minutes run, workout time

◯ Yes, all ◯ No, none ◯ Part of them

C. Badge: goals achieved, challenges completed

◯ Yes, all ◯ No, none ◯ Part of them

D. Daily food plan: list of things I've eaten

◯ Yes, all ◯ No, none ◯ Part of them

PEGASO
CHARACTER DESIGN STUDIO

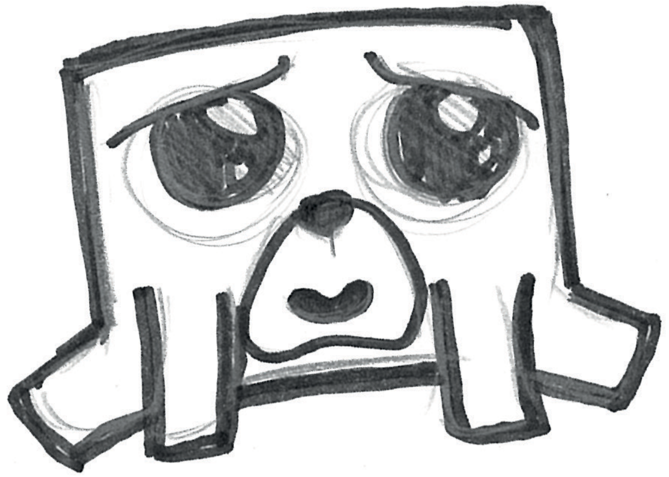

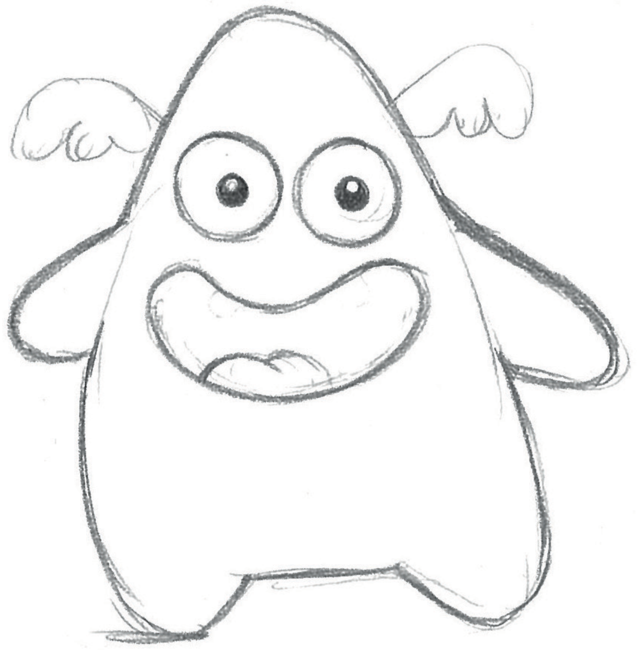

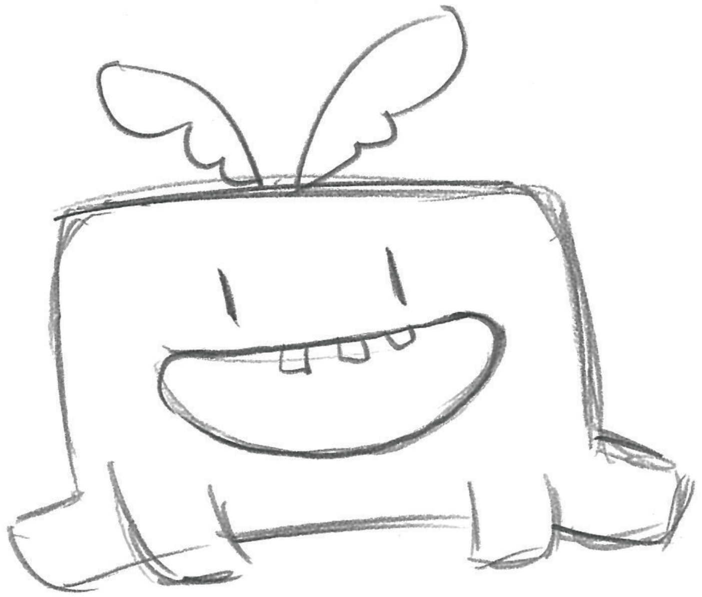

Check if you like it!

1. MASCOT

Pegaso is a cute and friendly monster/pet/alien who lives in the app to guide and entertain the user.

His simple and adaptive shape can be easily used in all merchandising activities.

RED HERRING 100 GLOBAL WINNER Gartner 2013 CoolVendor Neosperience

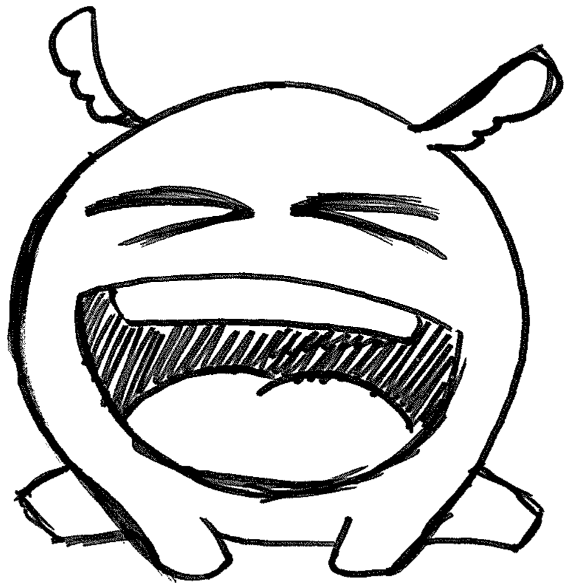

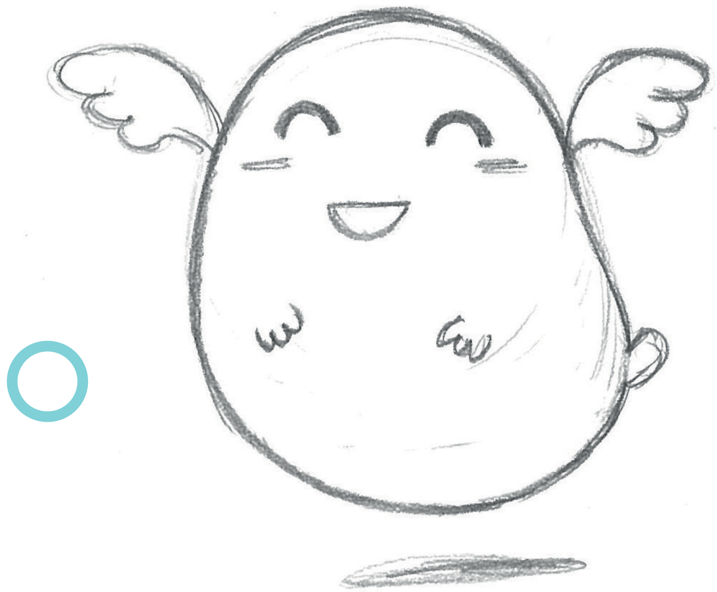

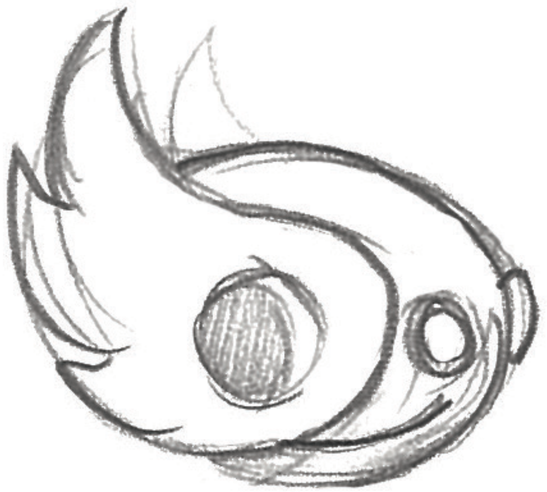

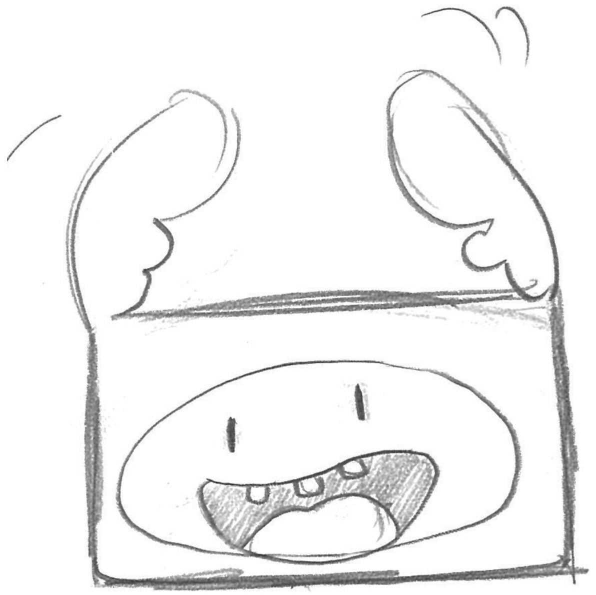

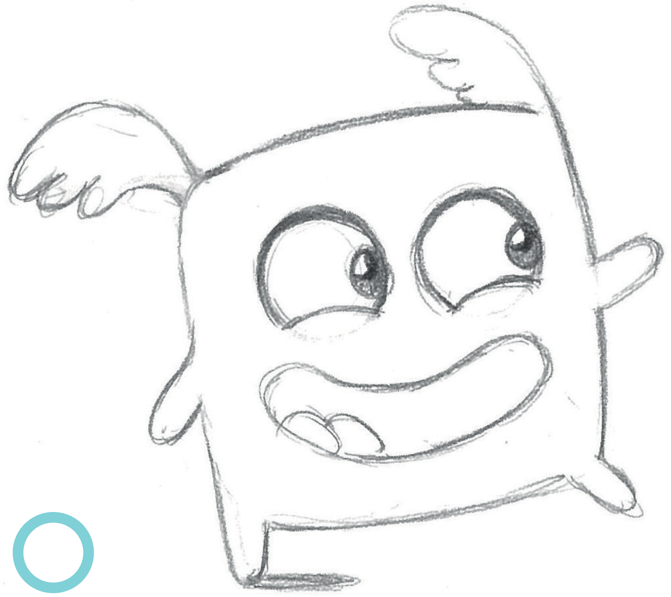

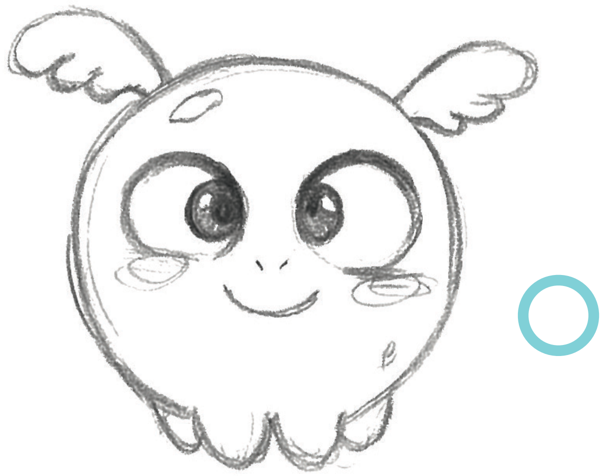

Check if you like it!

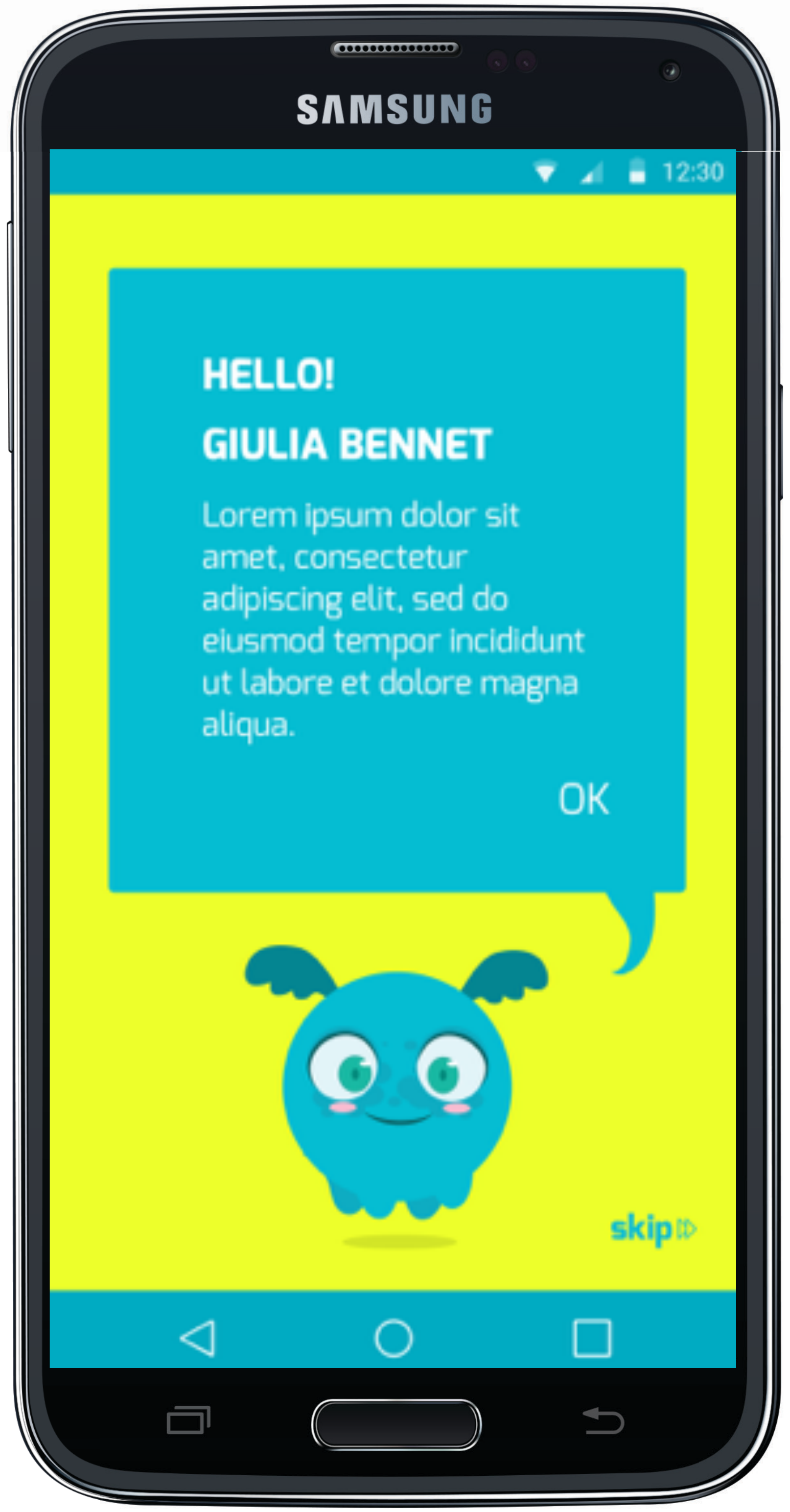

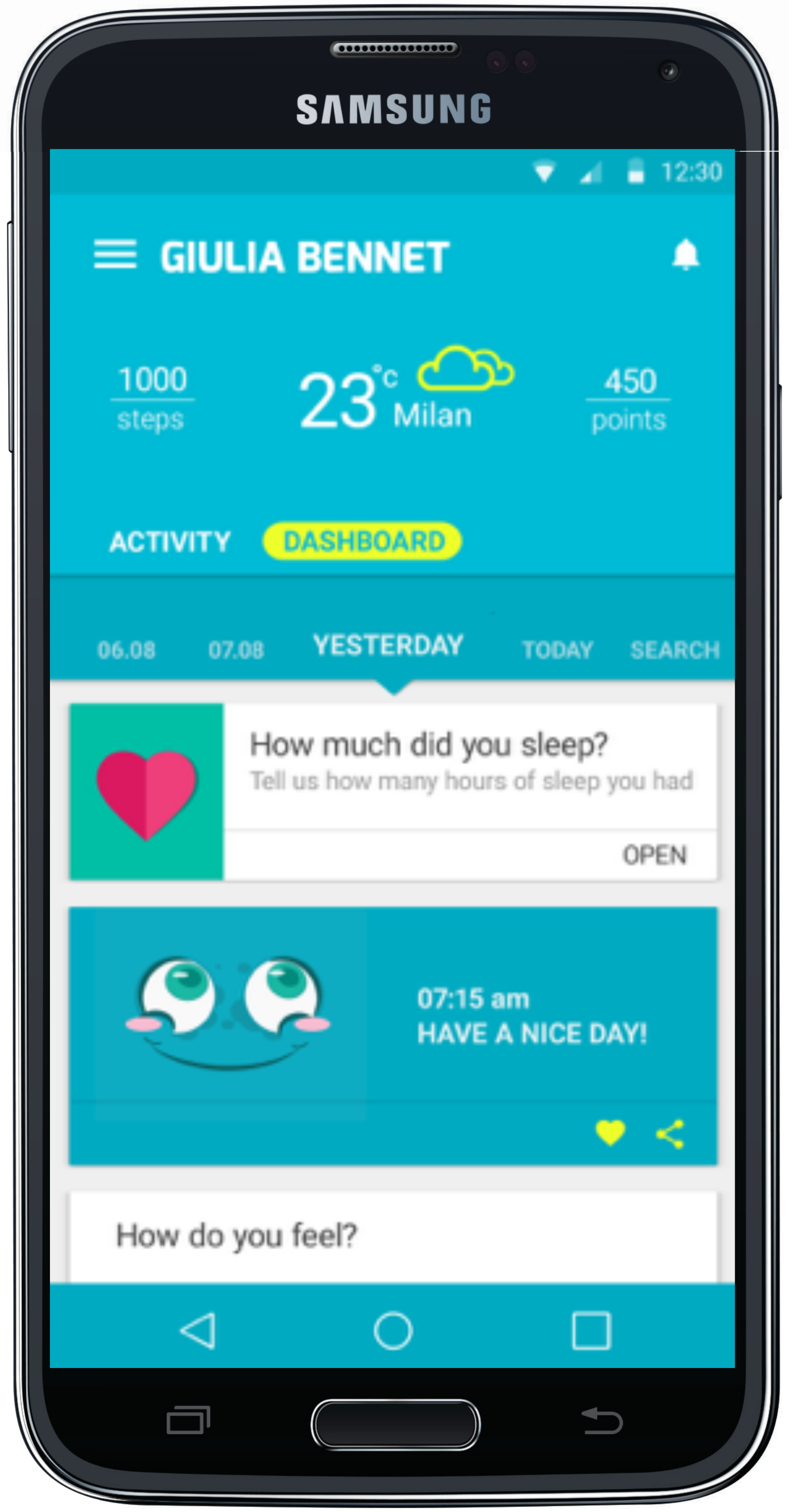

EXPRESSIVENESS

The mascot can change his shape or color to fit the app screen but is always recognizable for his strong expressiveness.

Do you like the idea of a mascot that follows you and gives you tips?

○ YES ○ NO

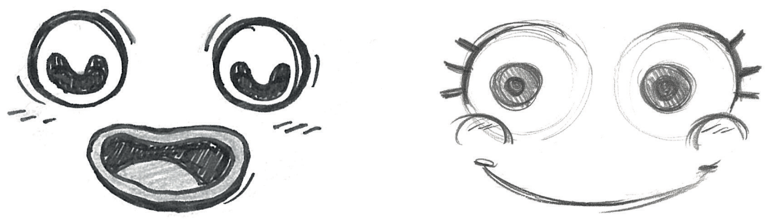

Check if you like it!

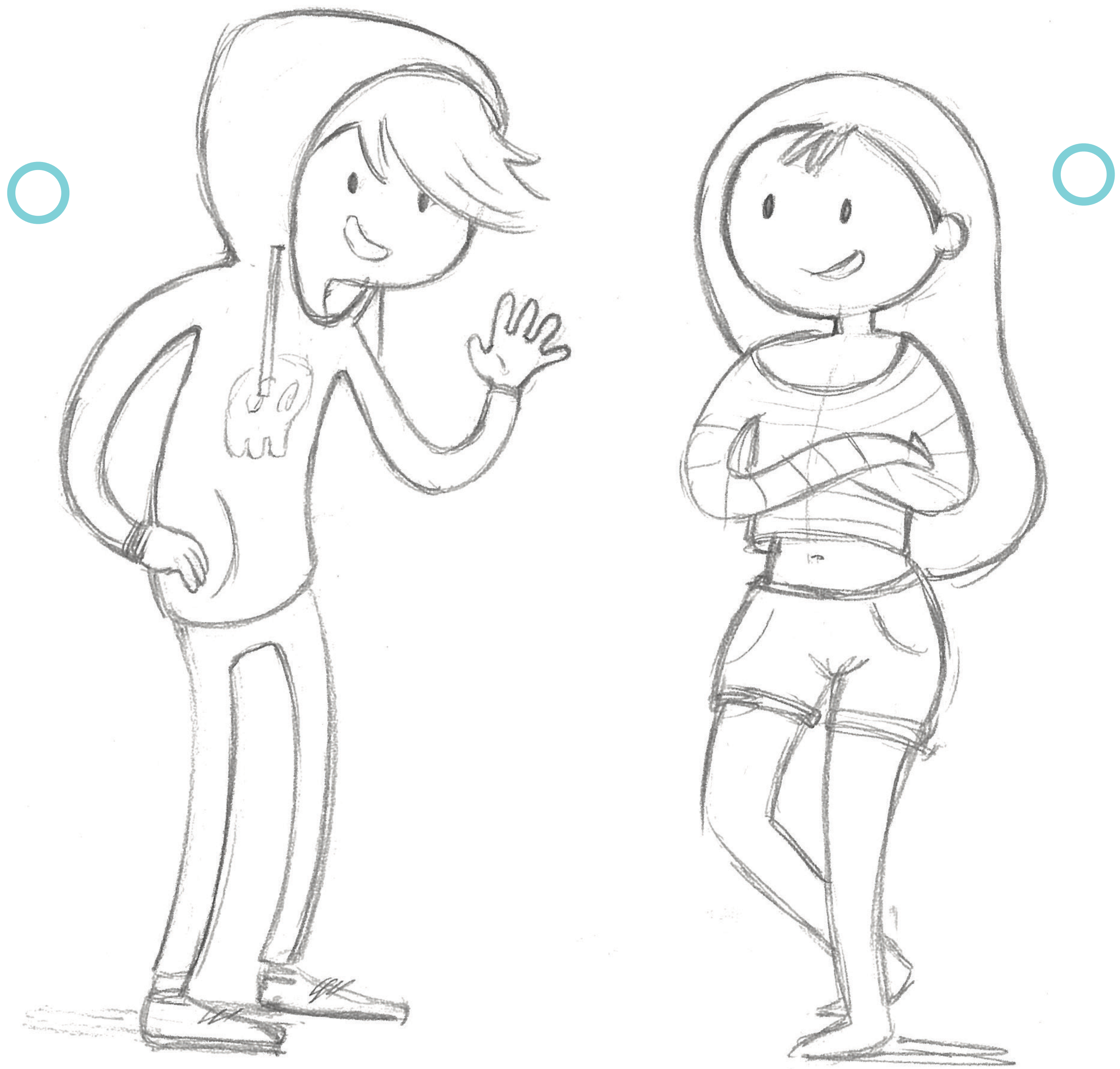

Check if you like it!

2. MY AVATAR

In the app the user can build or discover (after taking a quiz game) a cartoon character that will be his/her avatar in the Pegaso community.

Using the app consistently, users will unlock accessories and other details to customize the avatar.

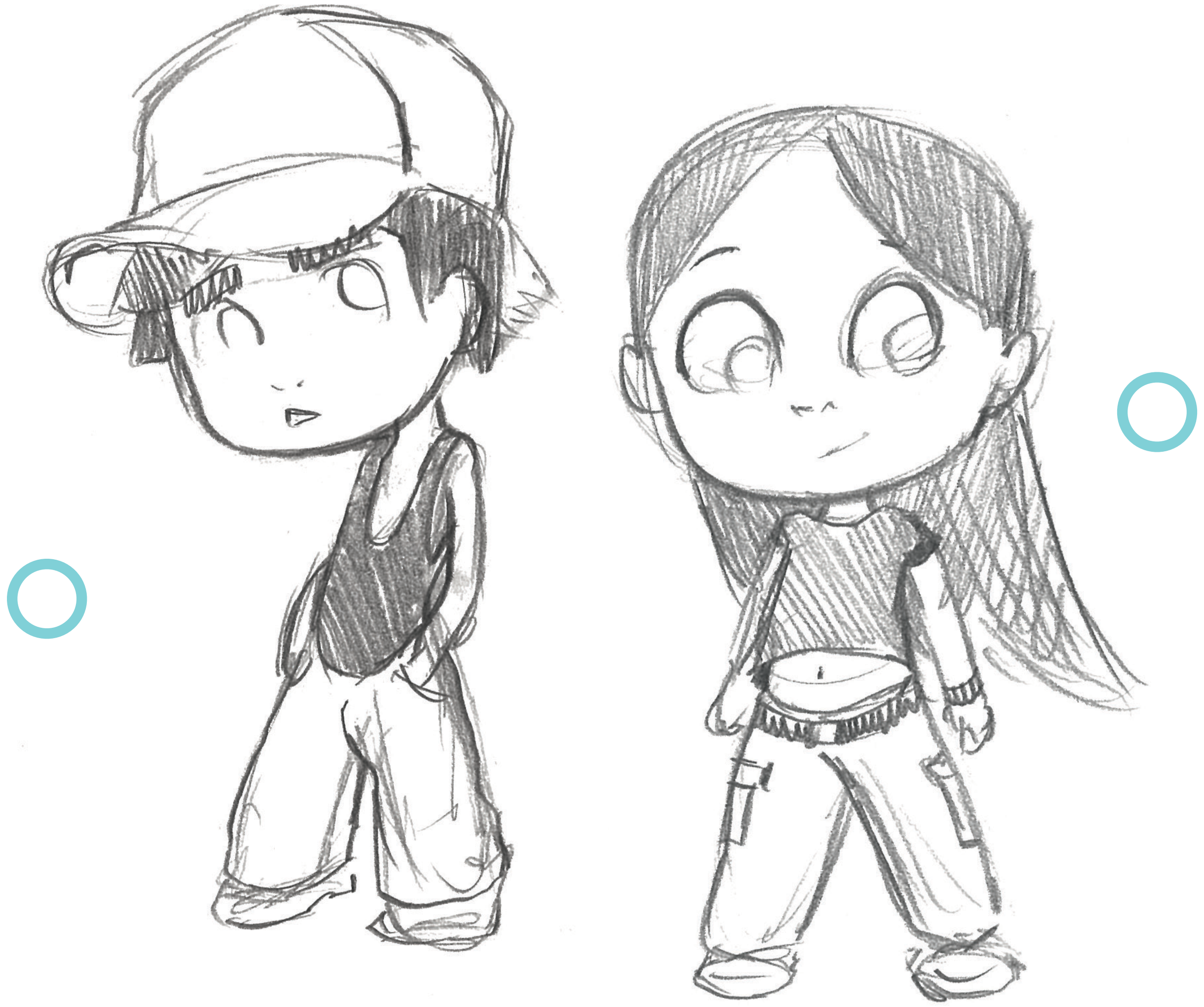

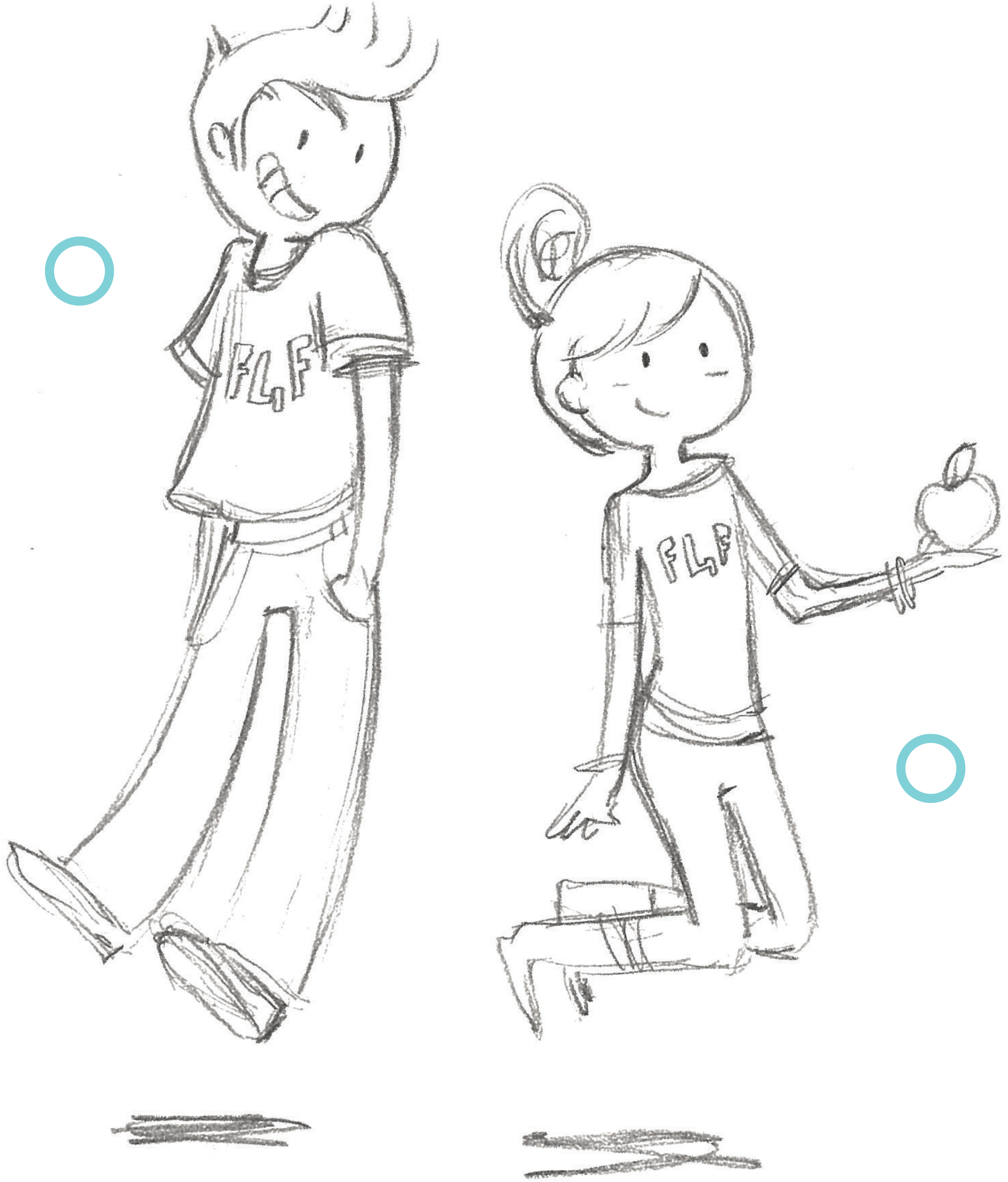

Check if you like it!

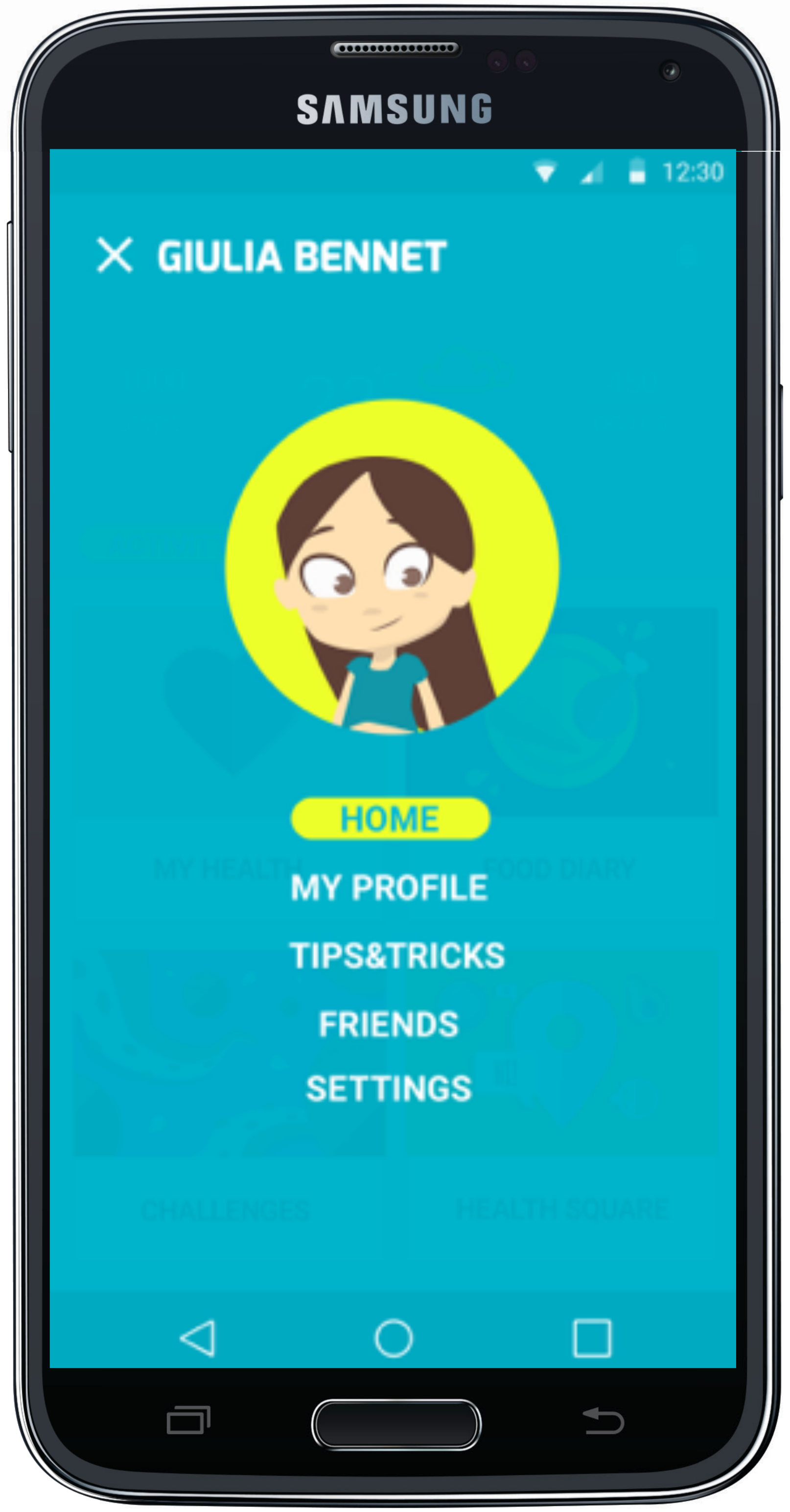

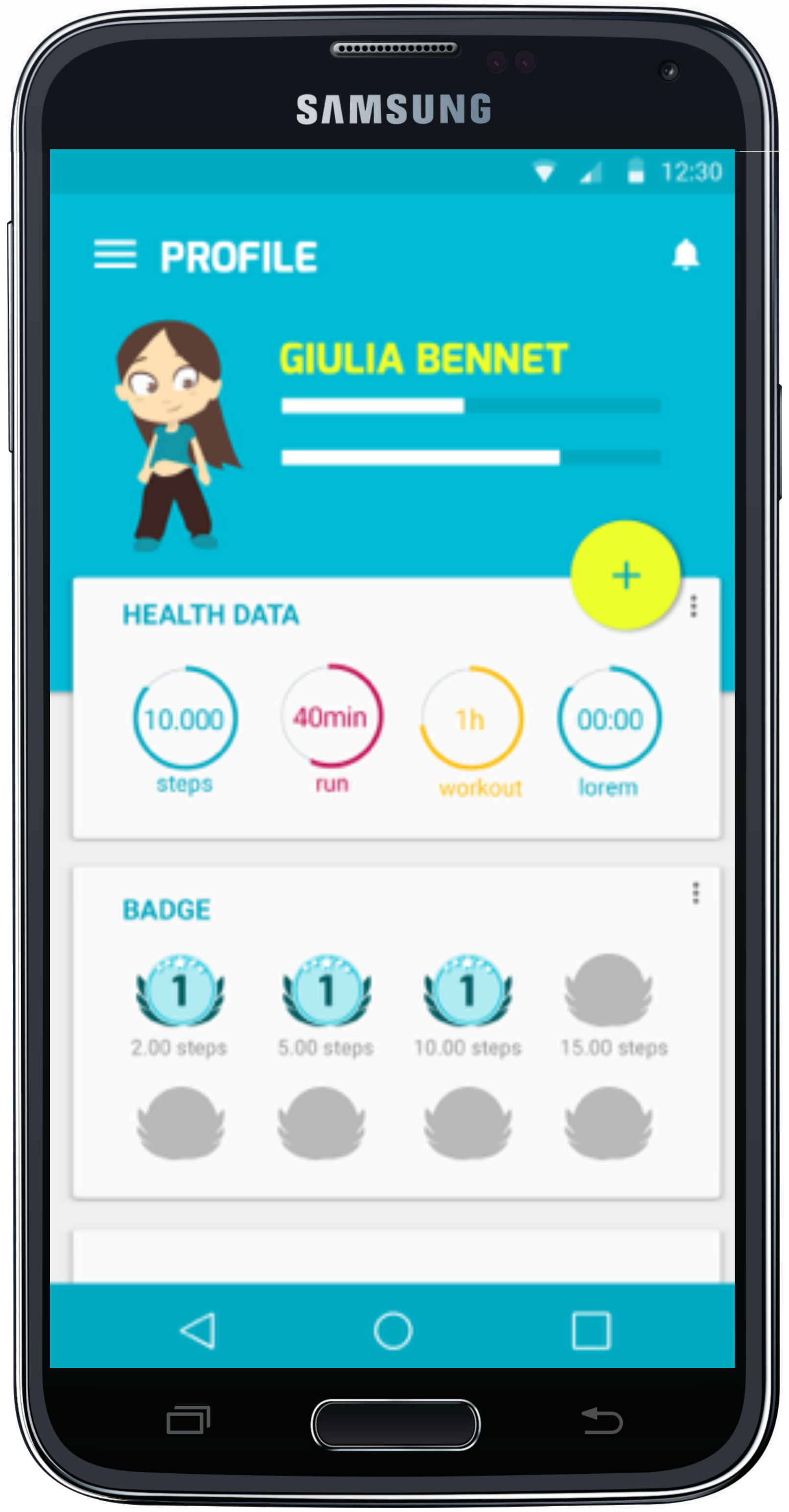

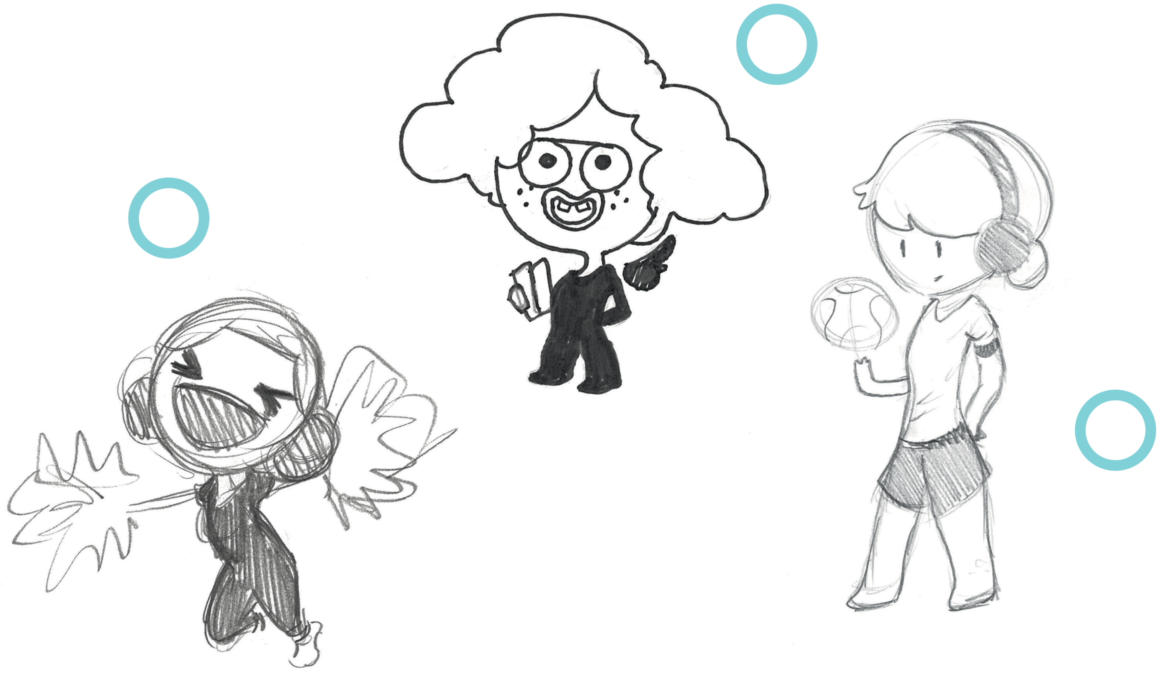

AVATAR

An alternative way to personalize your profile is building an avatar.
Do you like the idea or prefer the photo?

○ AVATAR ○ PHOTO

Check if you like it!

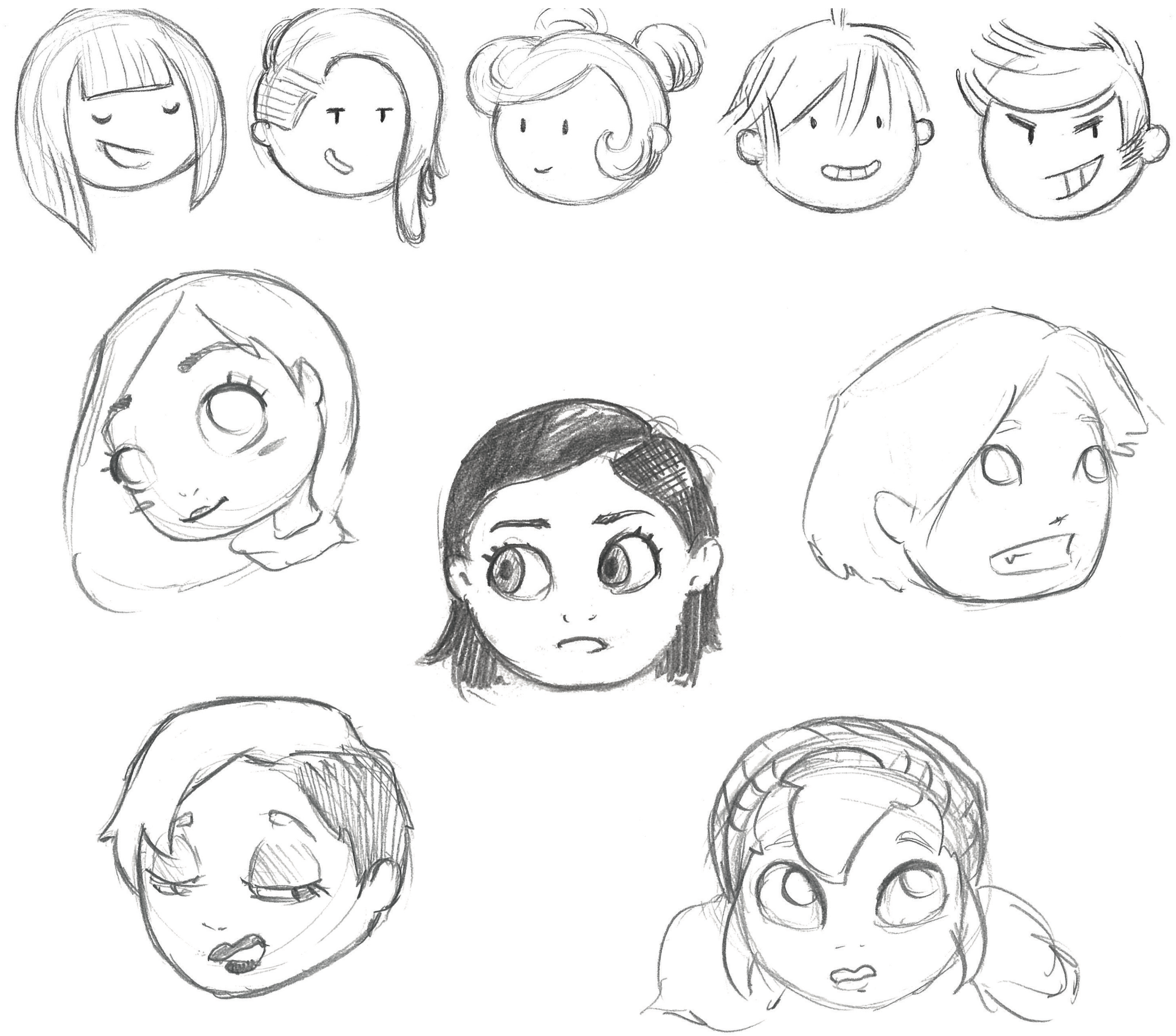

Check if you like it!

Avatars in Pegaso chat

A smaller version of the avatar can be used to chat with other members of the community.

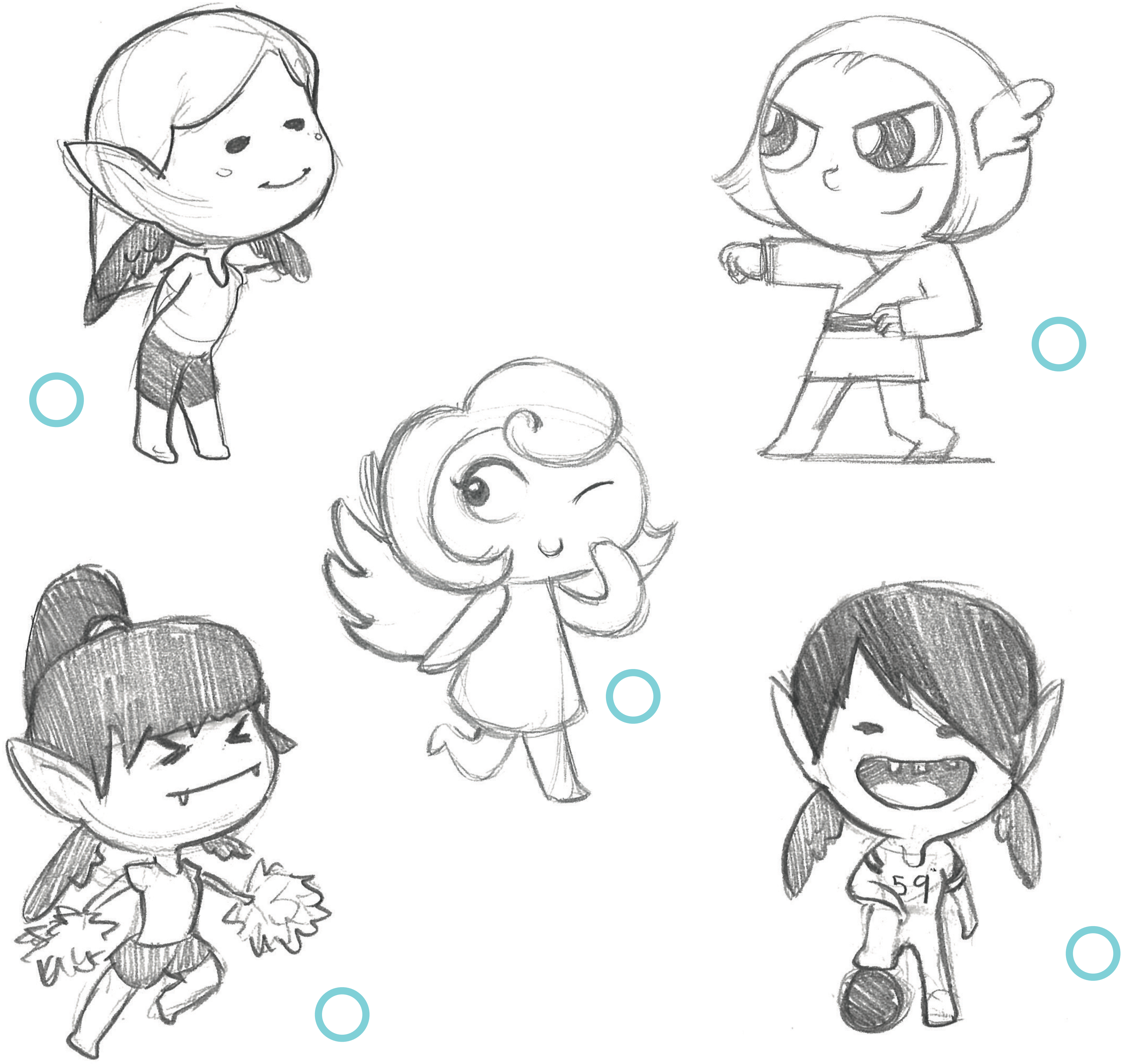

Check if you like it!

3. FANTASY TRAINER

A little tribe of winged creatures will guide the user in games and challenges.

Each creature is specialized in a sport activity or health matters.

The little creatures can be also used as game characters.

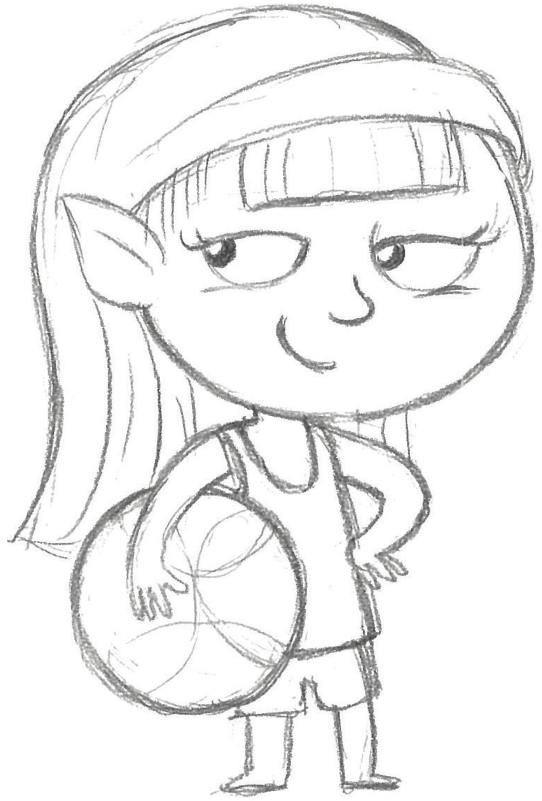

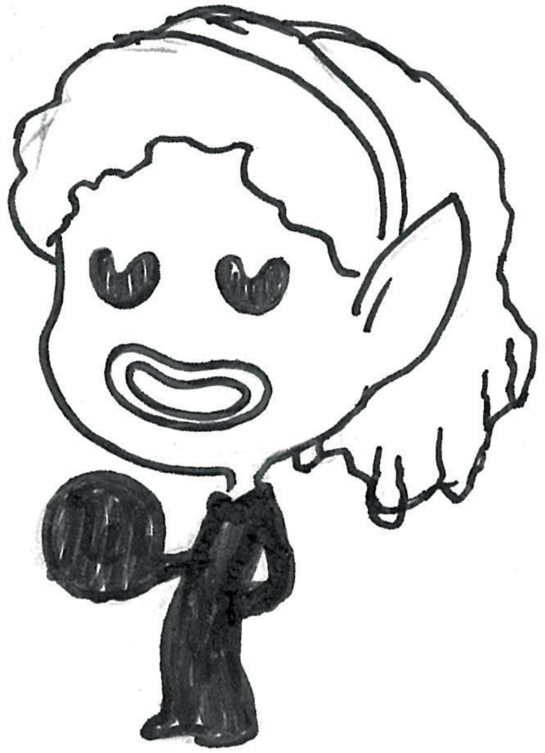

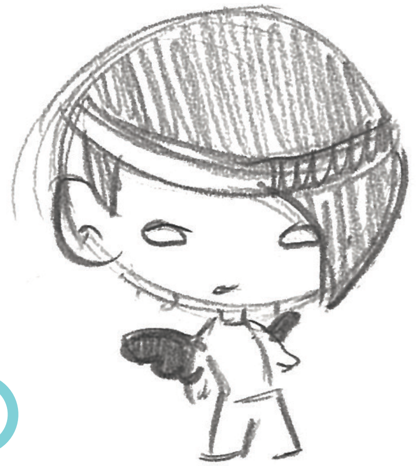

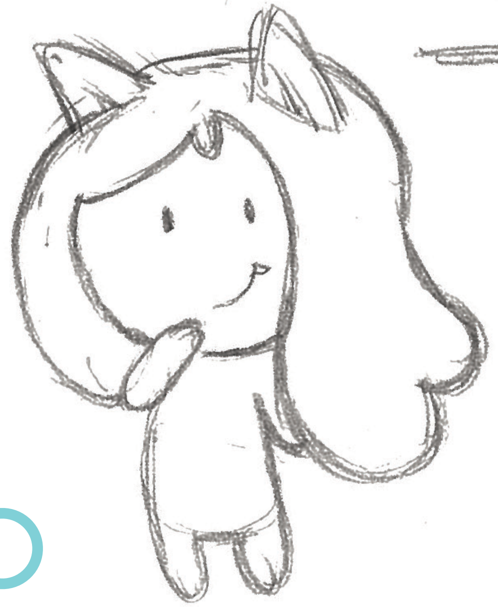

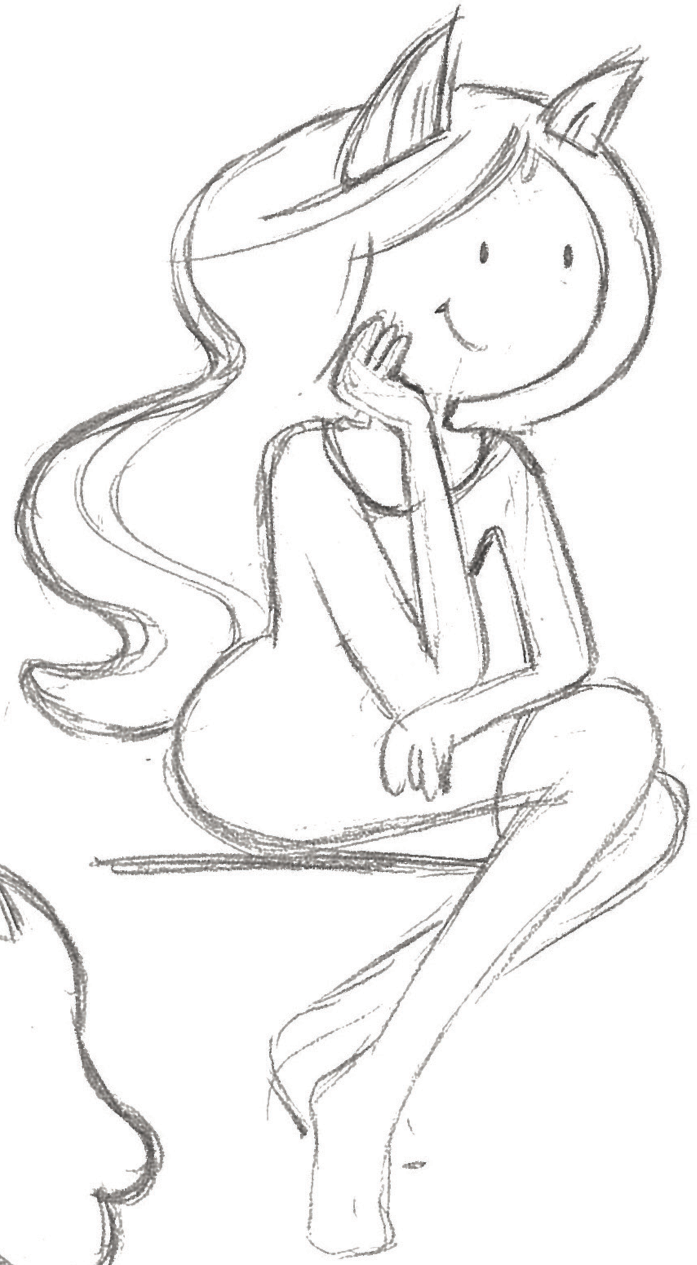

Check if you like it!

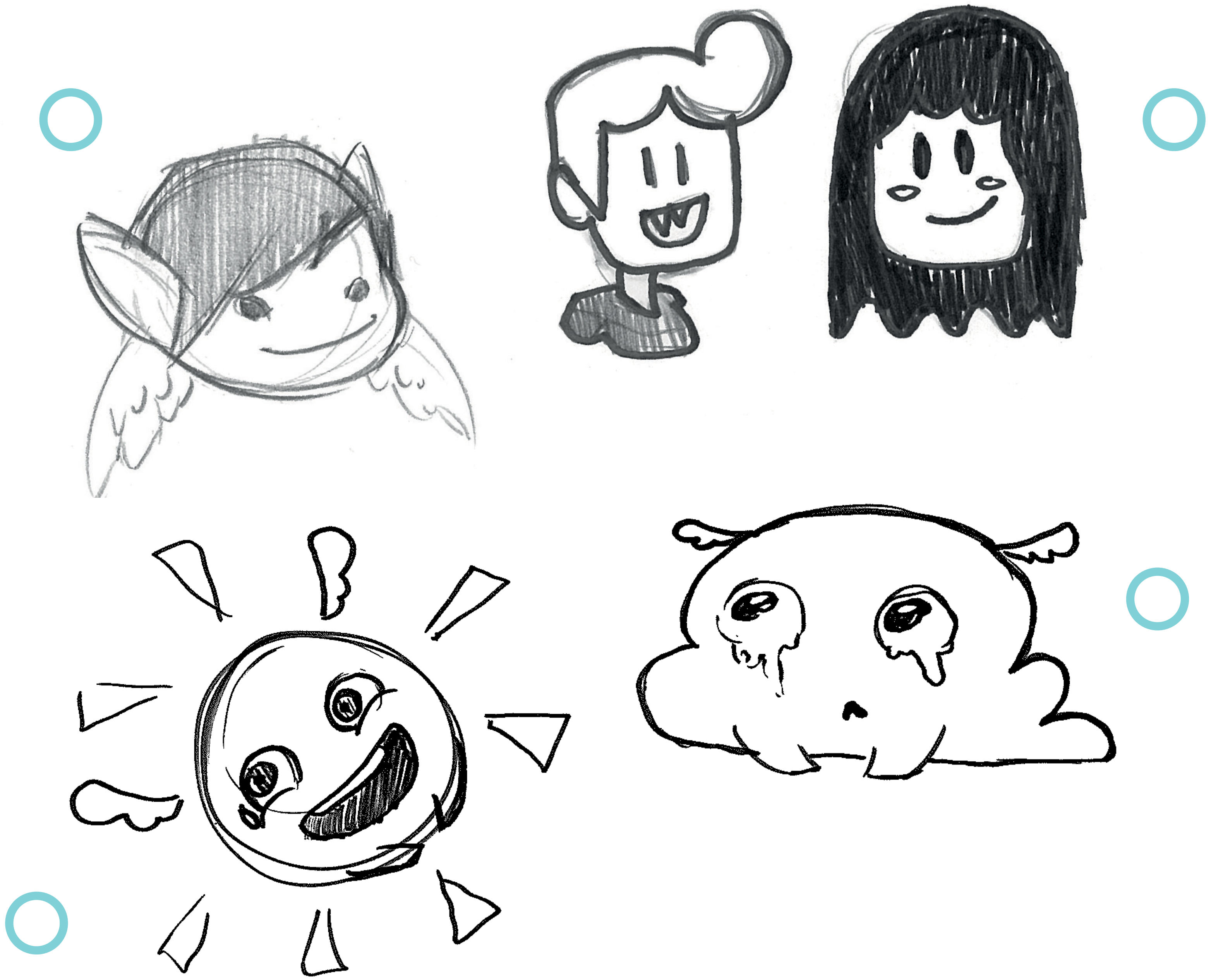

Check if you like it!

4. EMOTICONS

A complete set of emoji and stickers for chat and meme.

Do you like the idea to have a set of emoji and stickers exclusive to PEGASO users?

◯ YES ◯ NO

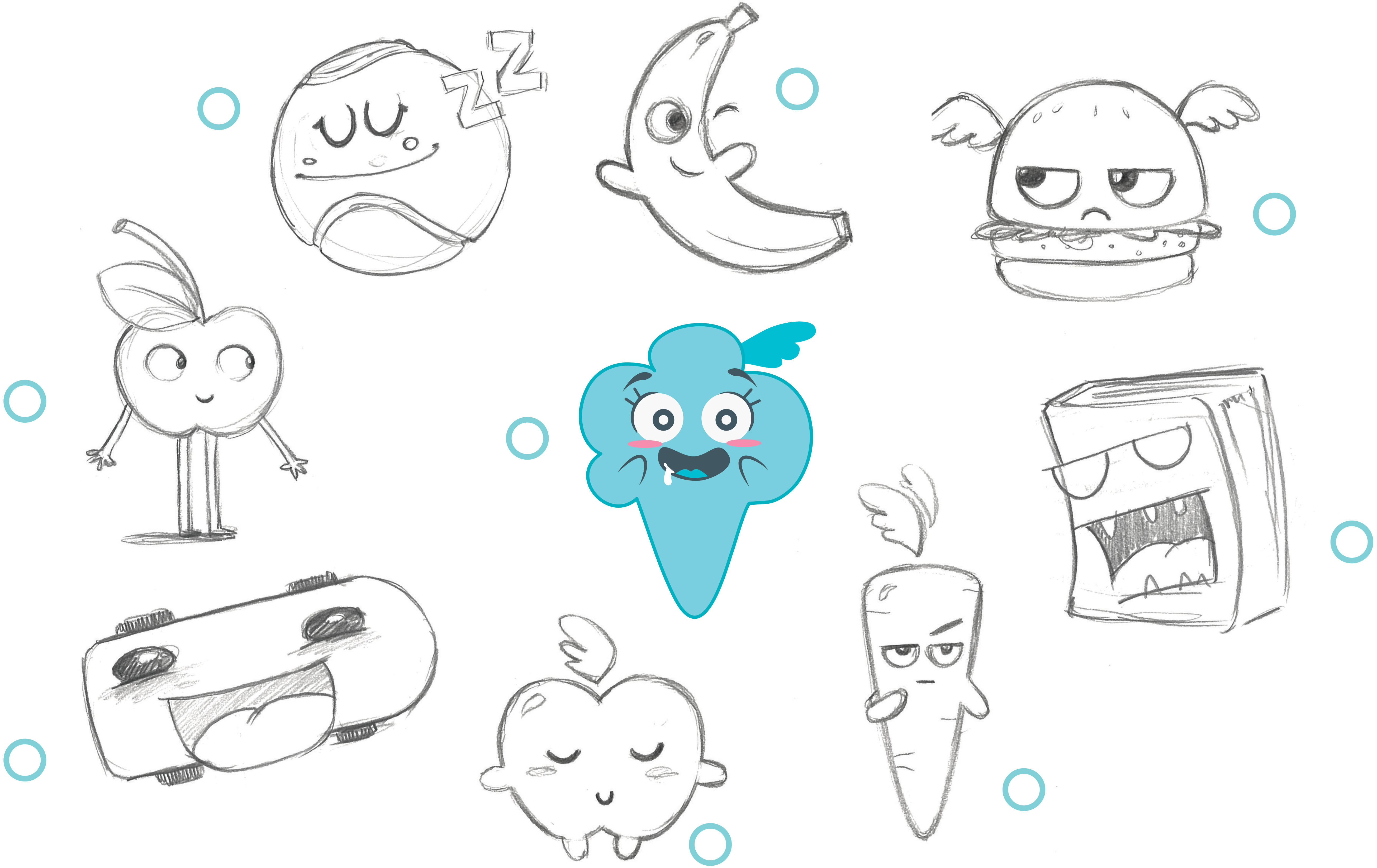

RED HERRING 100 WINNER GLOBAL
Gartner 2013 Cool Vendor
Neosperience

PEGASO
Food Diary

We ask the participants to test the comprehension of the icons of Food Diary section. These icons represent different food categories. Do you recognize them? Write the meaning next to the icon.

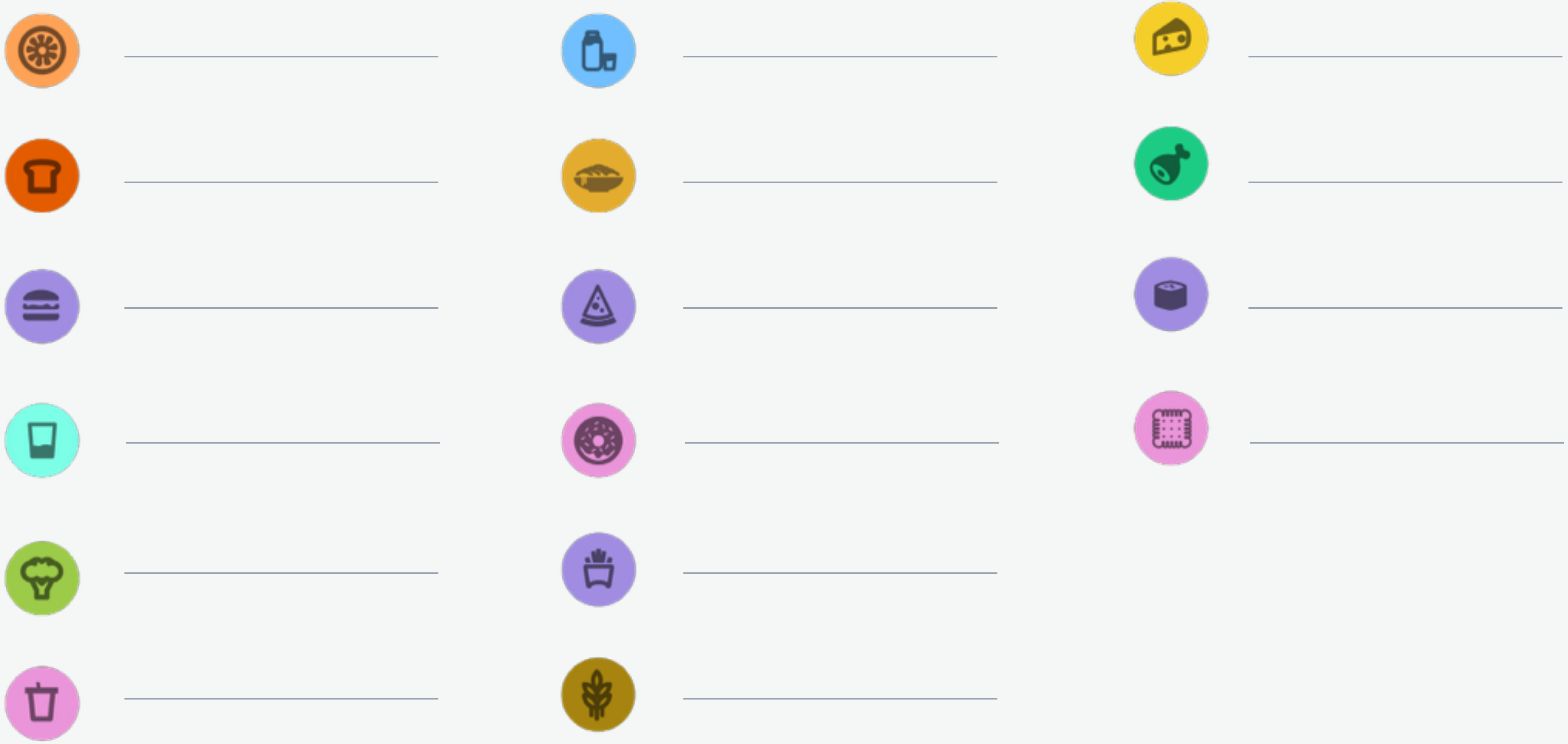

RED HERRING GLOBAL 100 WINNER
Gartner 2013 CoolVendor
Neosperience

OVERVIEW

The first step of the food diary requires that the app will monitor user's food behavior for 15 days, to set specific goals. For example, if behavior and habits show that the user does not eat vegetables, the goal will be to include them in the daily diet. The (+) button will open a pop up with all foods (yellow screen in the next slide). Look also at the screens in the next 2 slides before answering the questions.

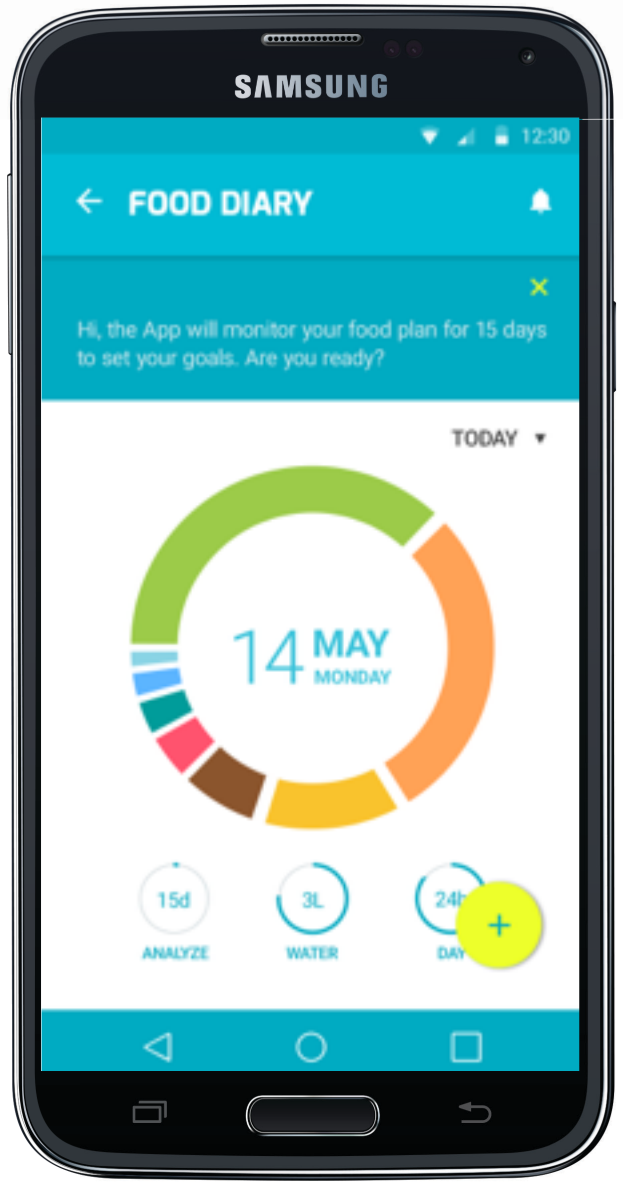

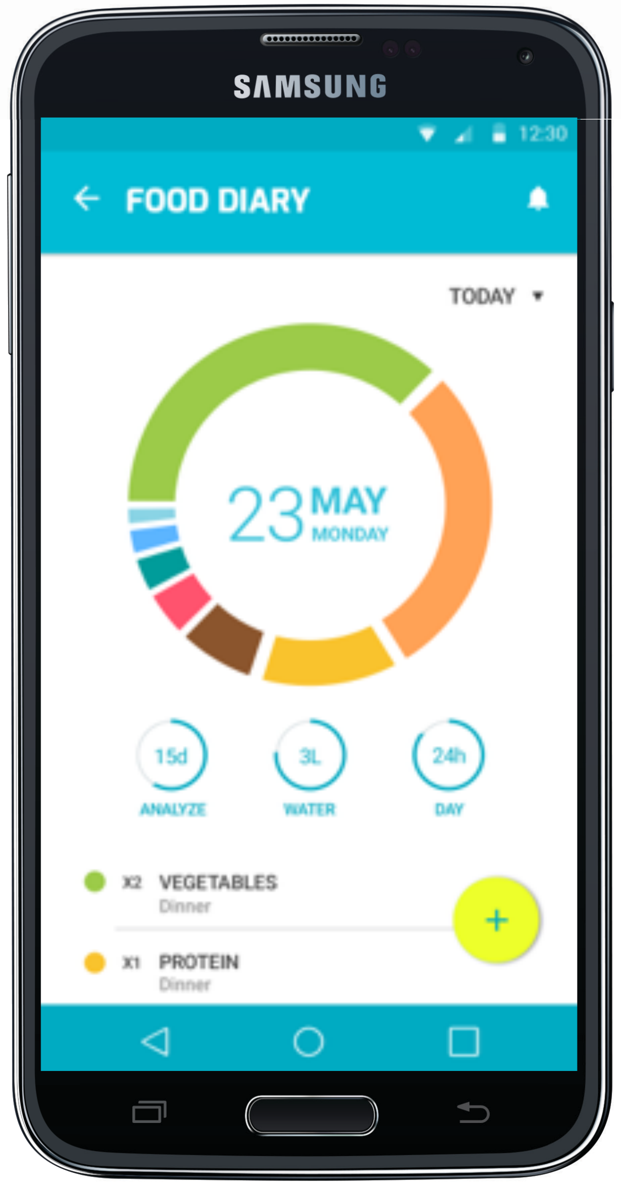

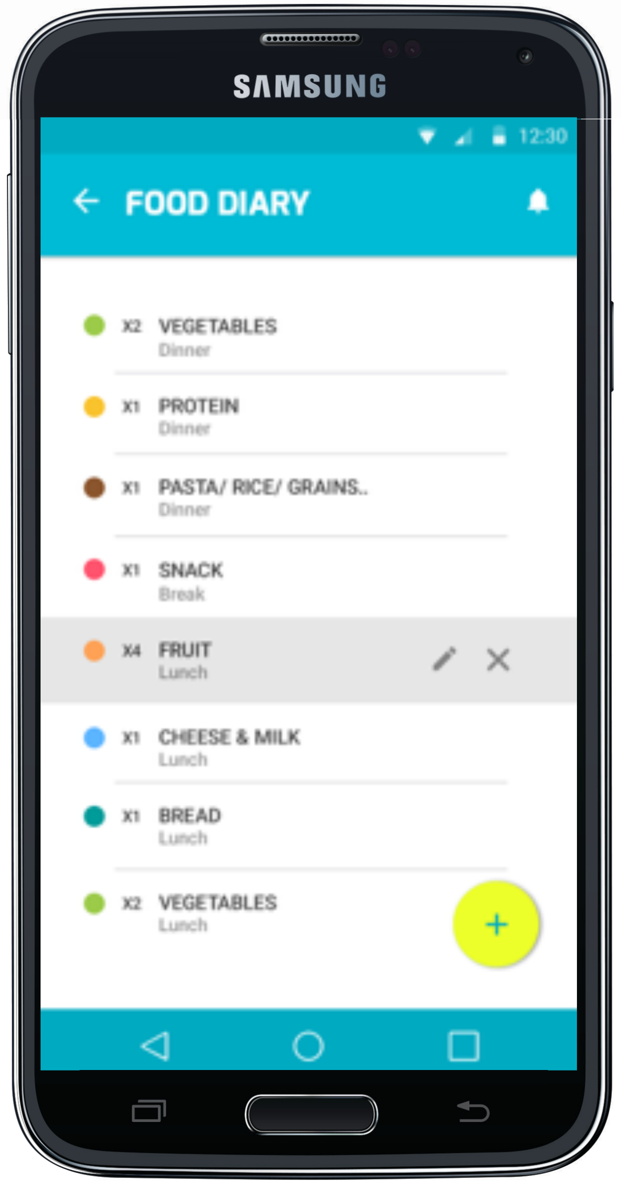

These screens show the monitoring section.

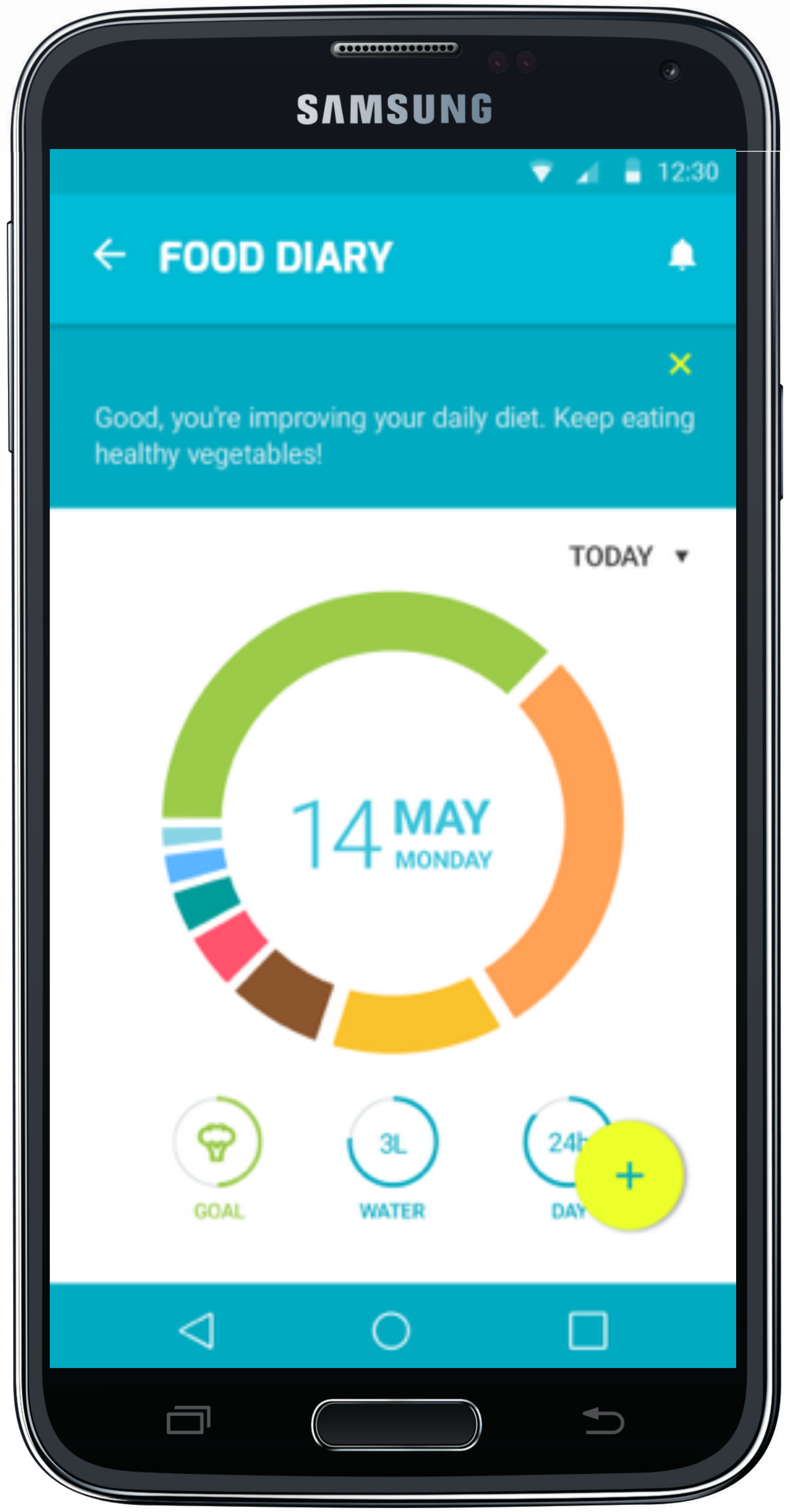

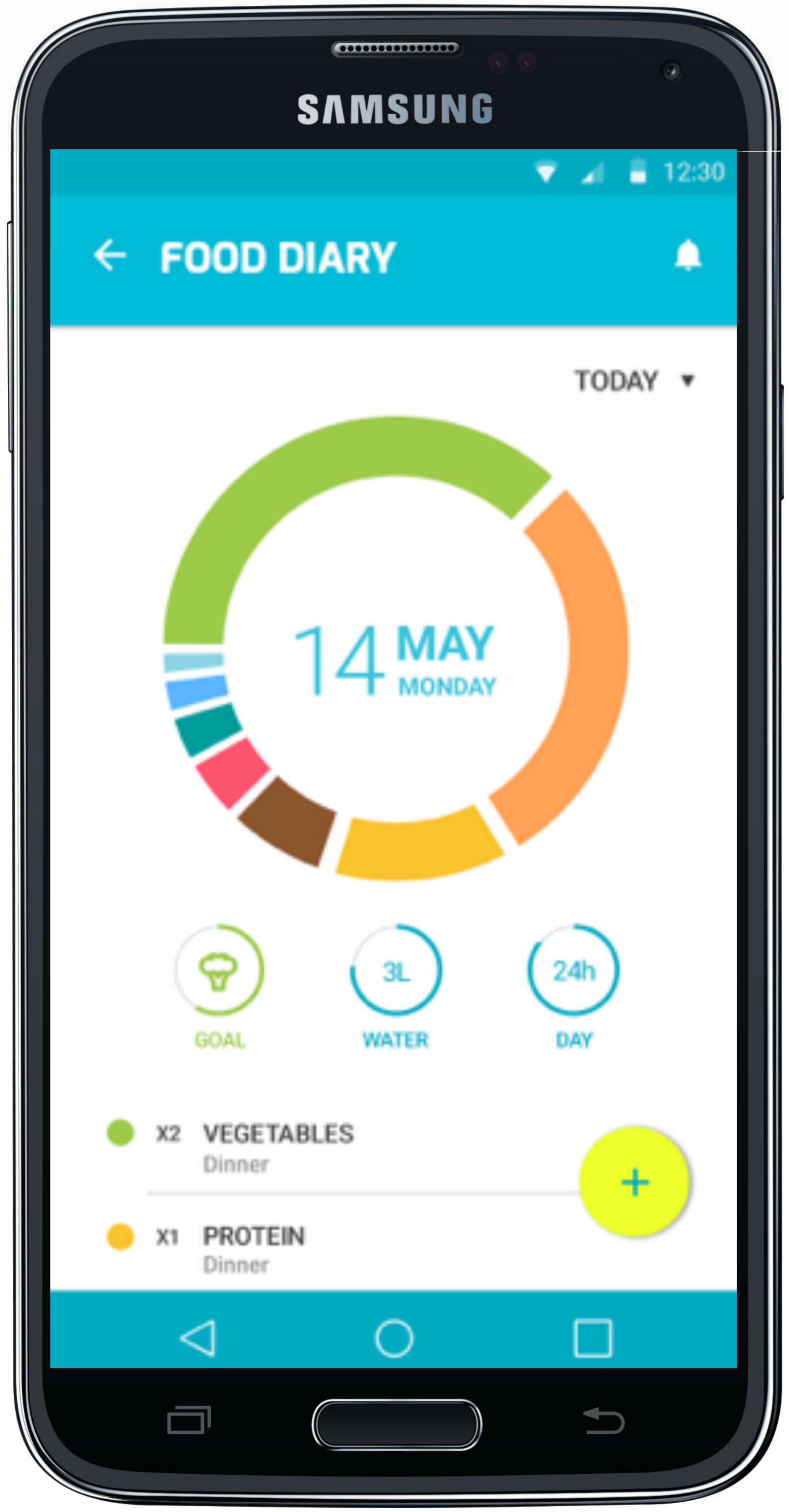

This screen shows the food diary at the end of the monitoring process.

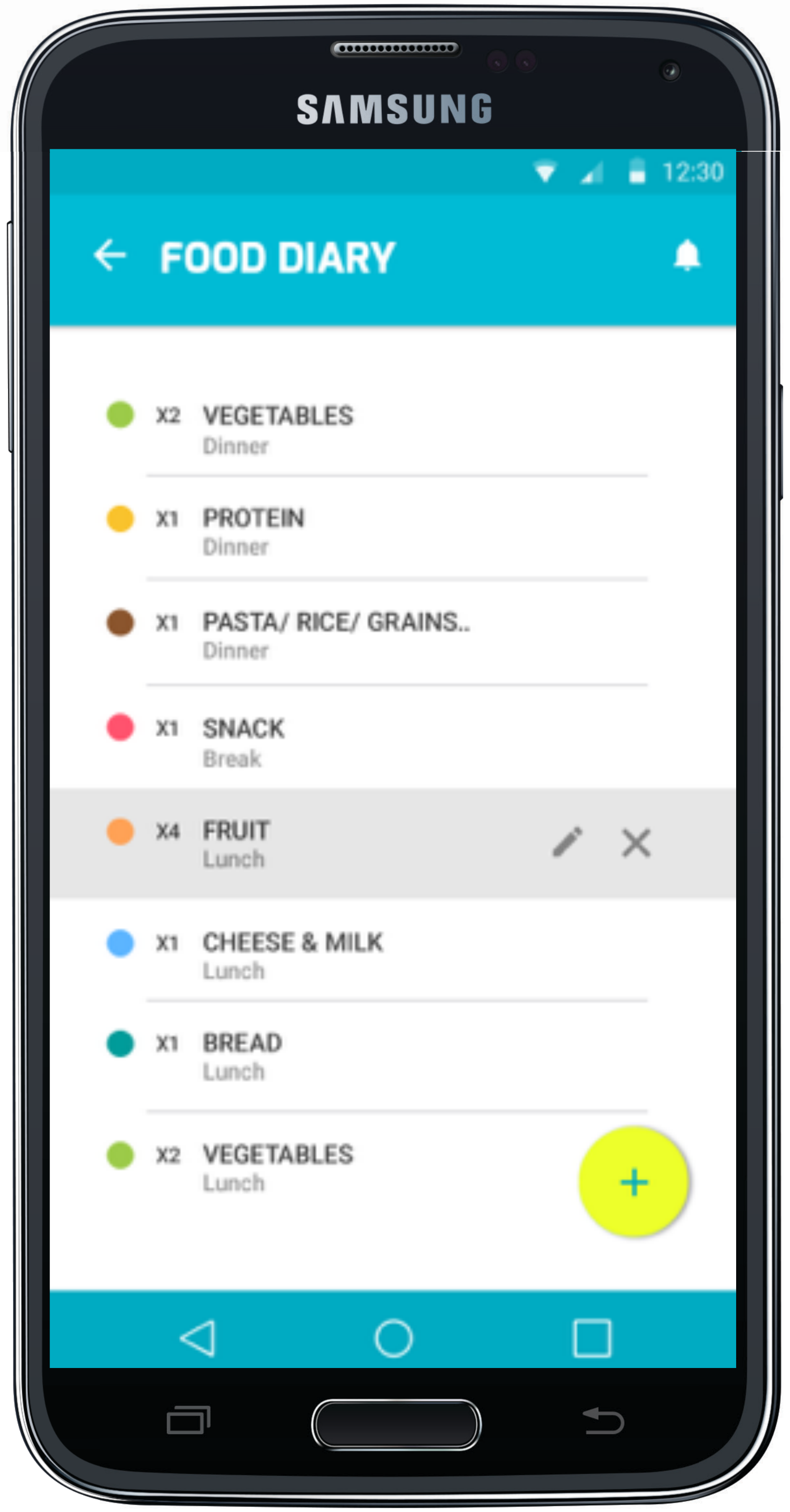

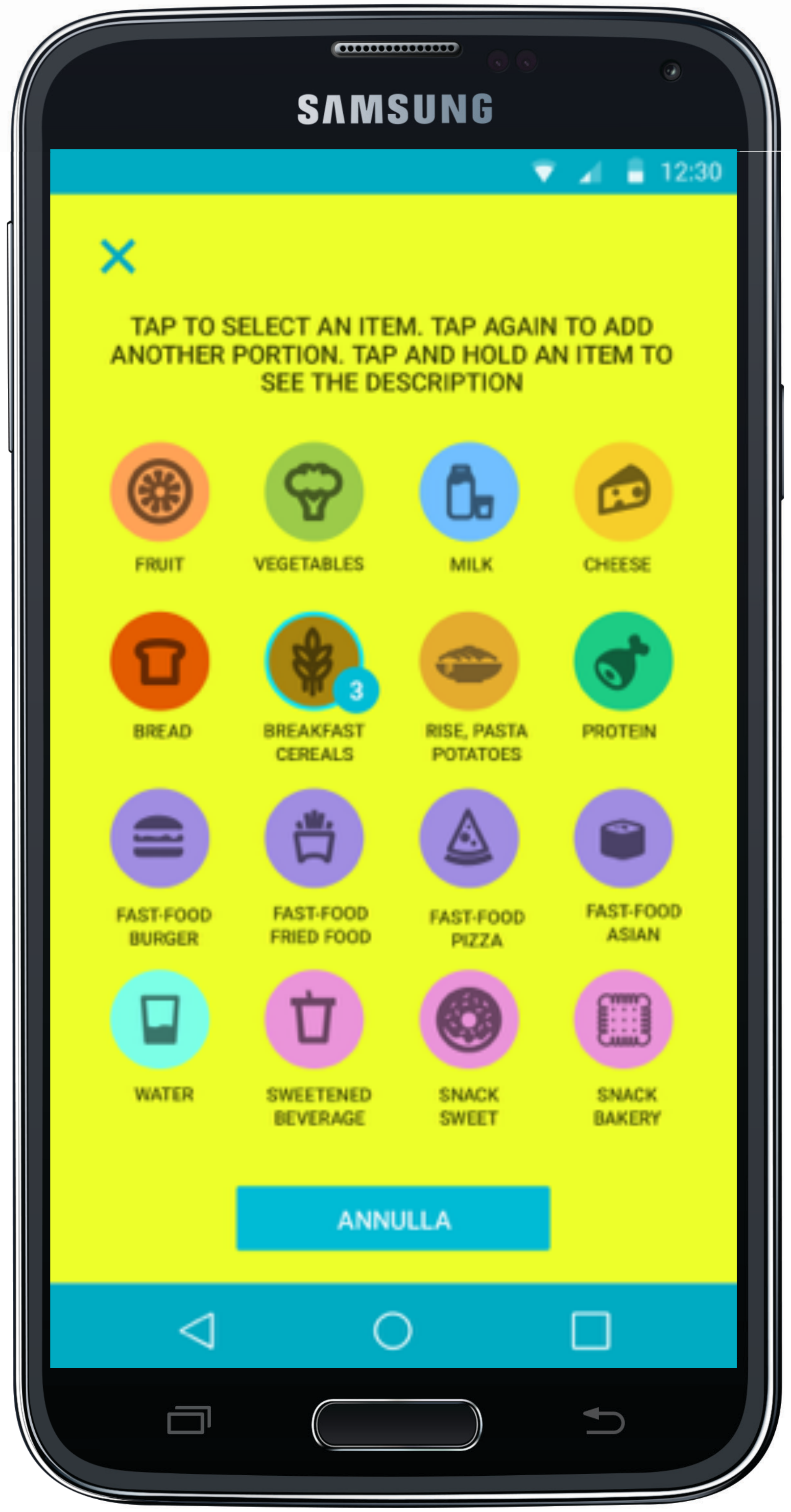

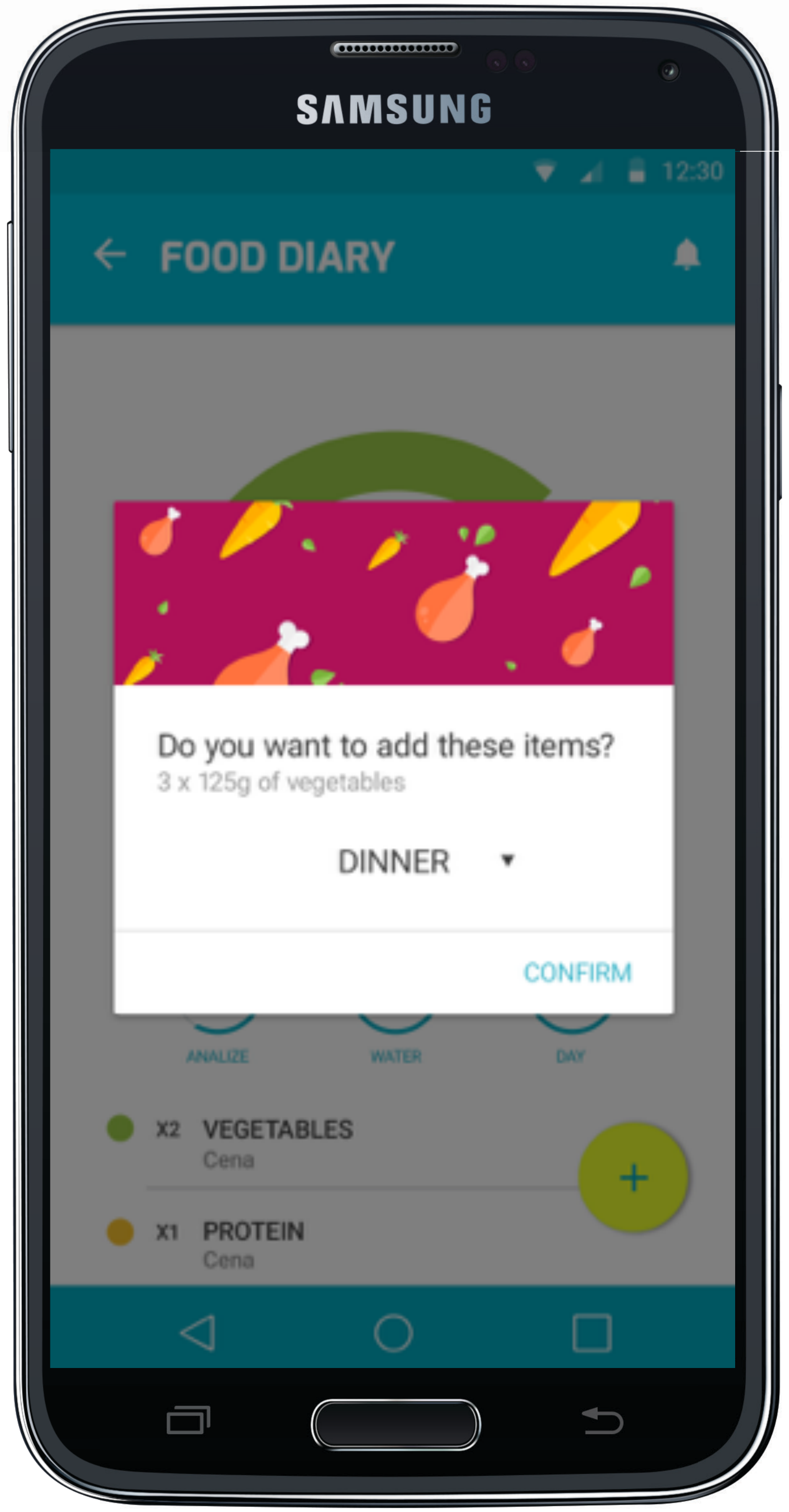

Neosperience

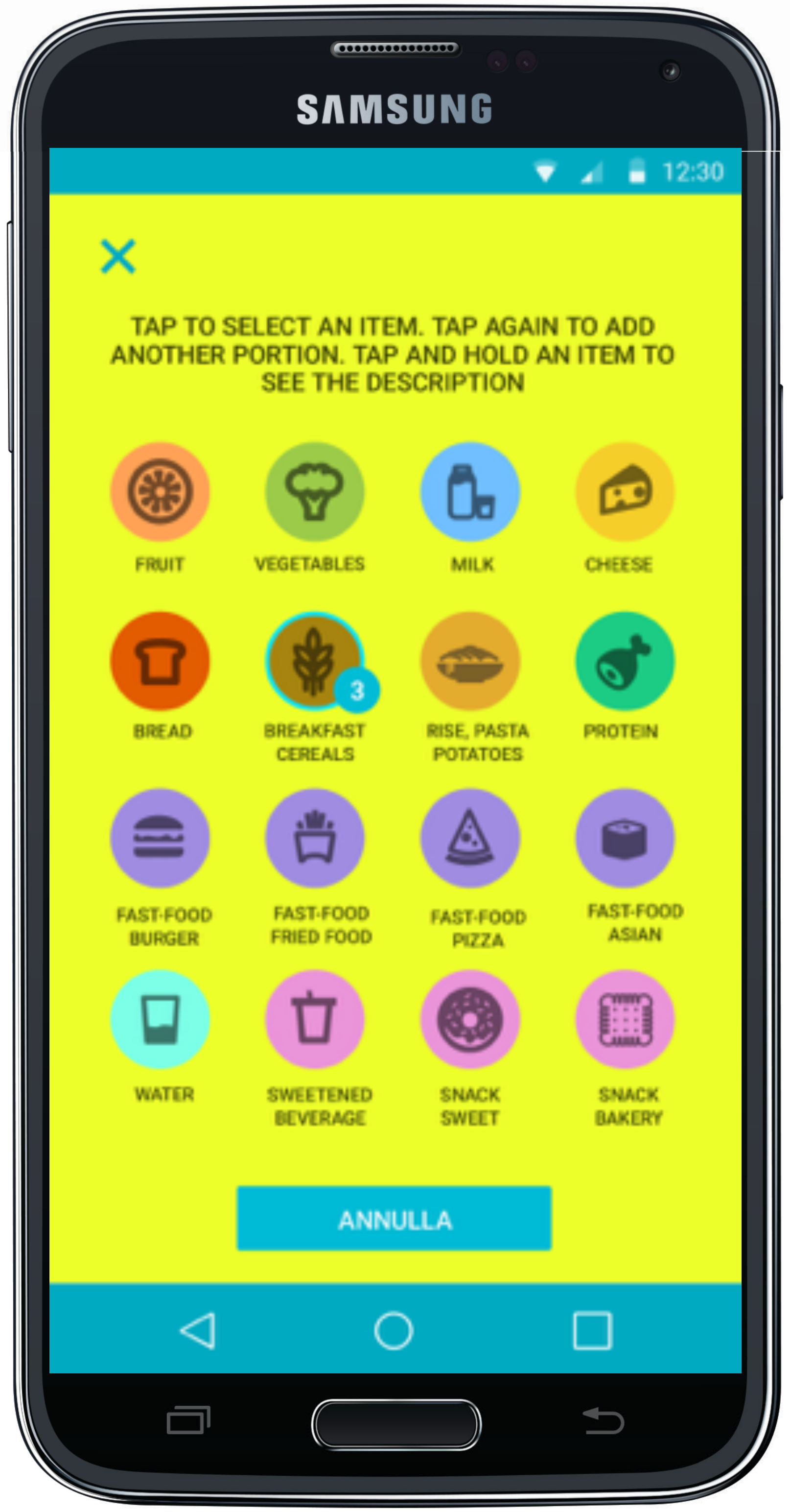

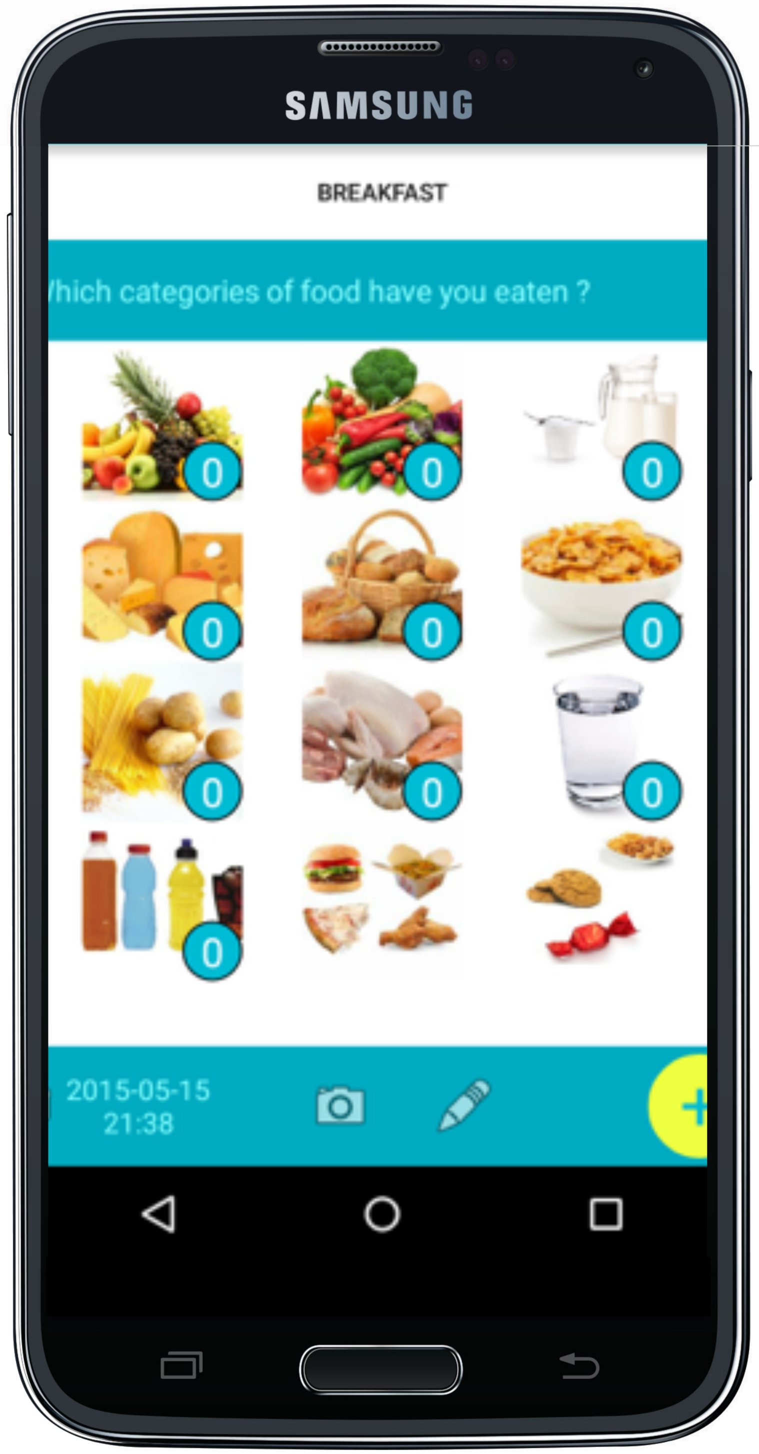

Check the one that you prefer

Check the one that you prefer

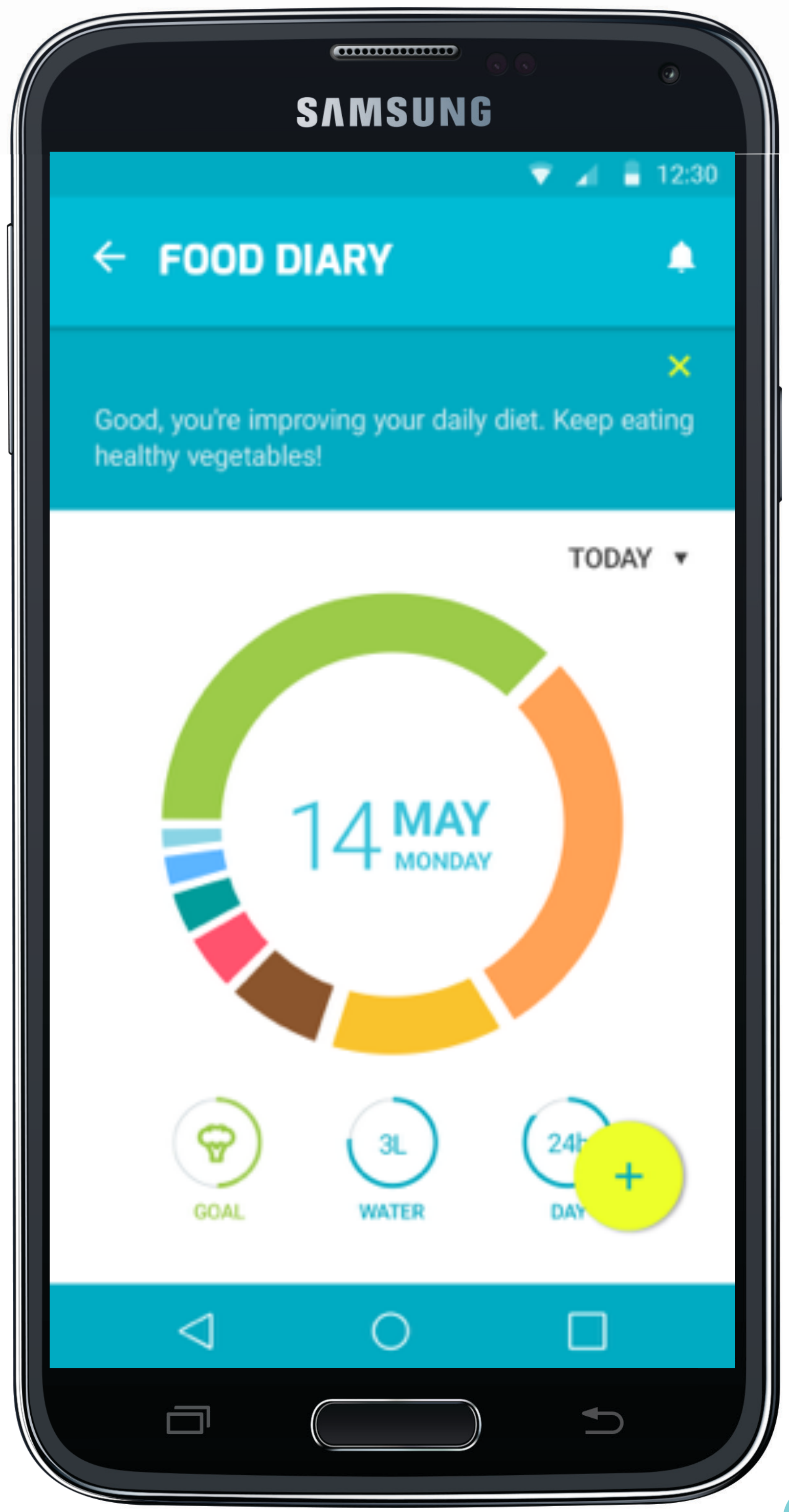

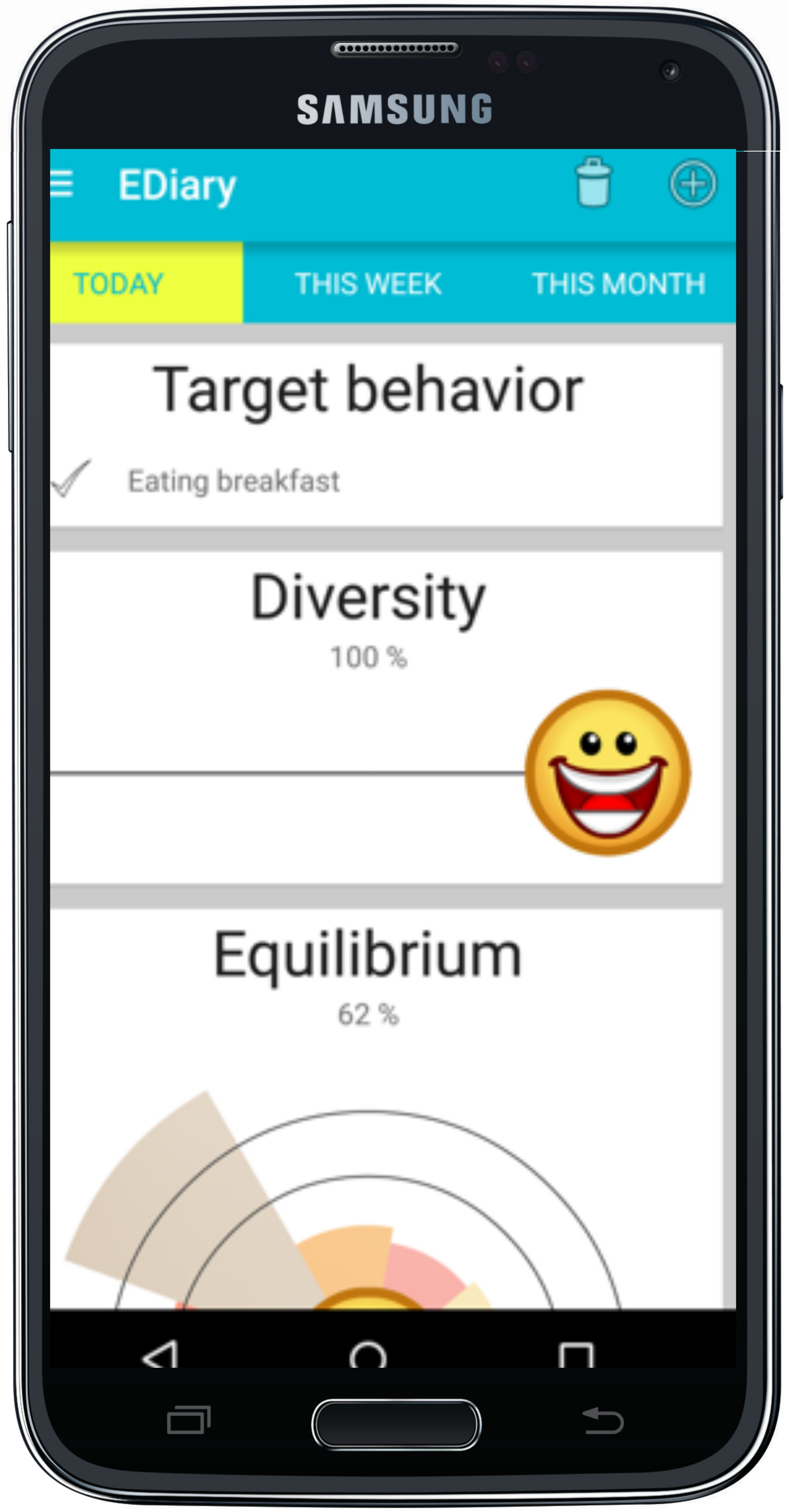

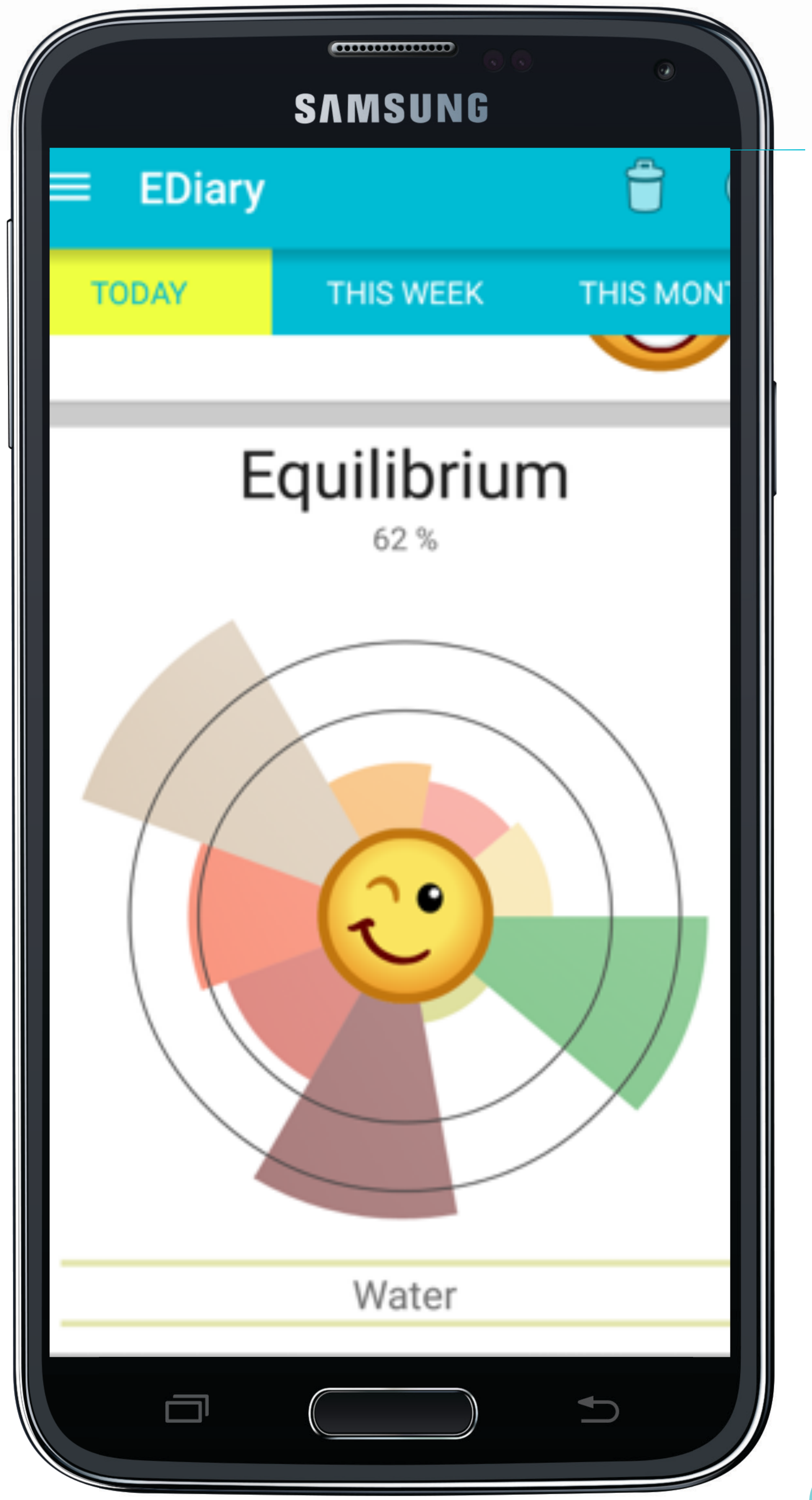

PEGASO
PLACE DISCOVERER

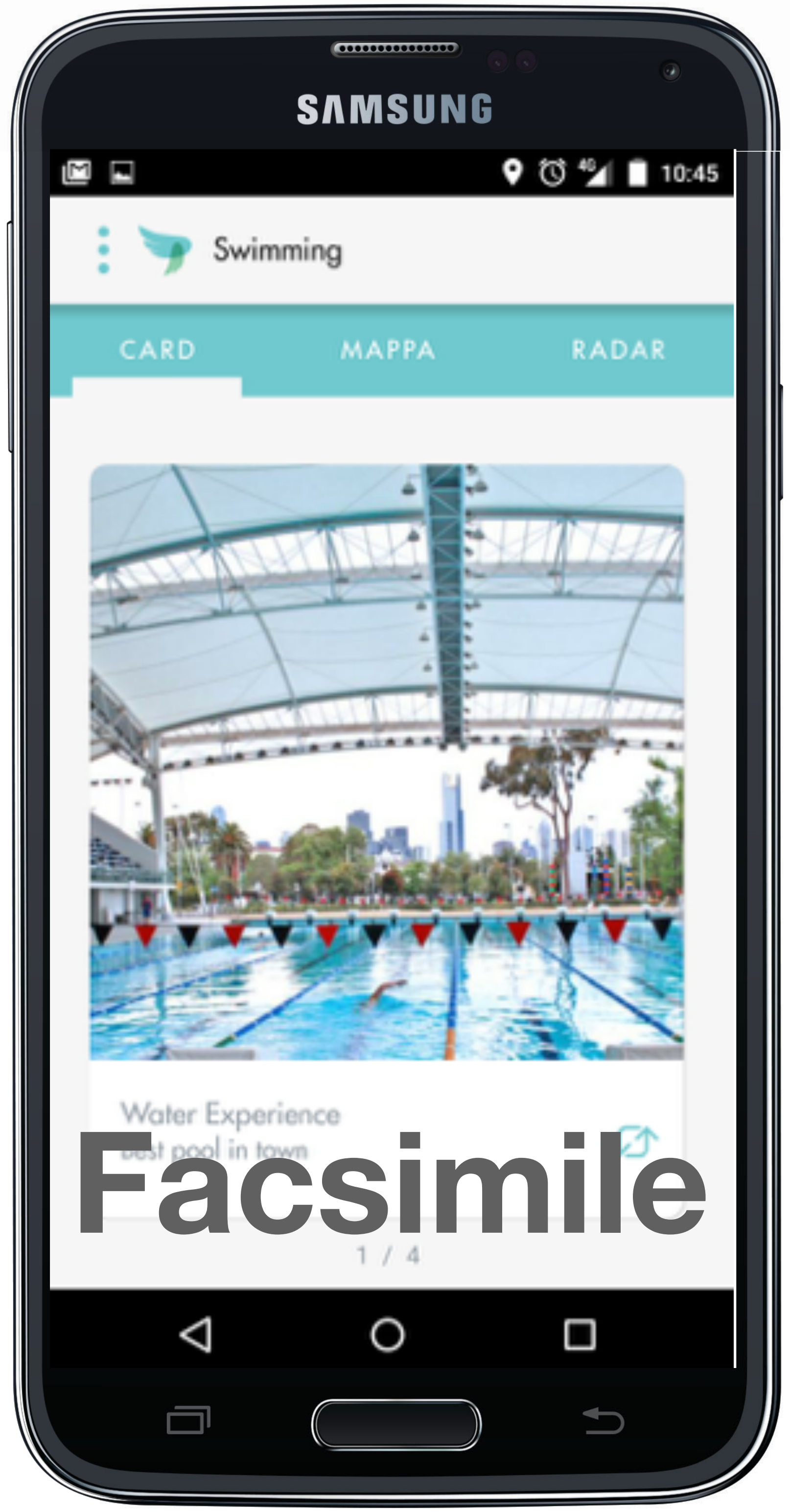

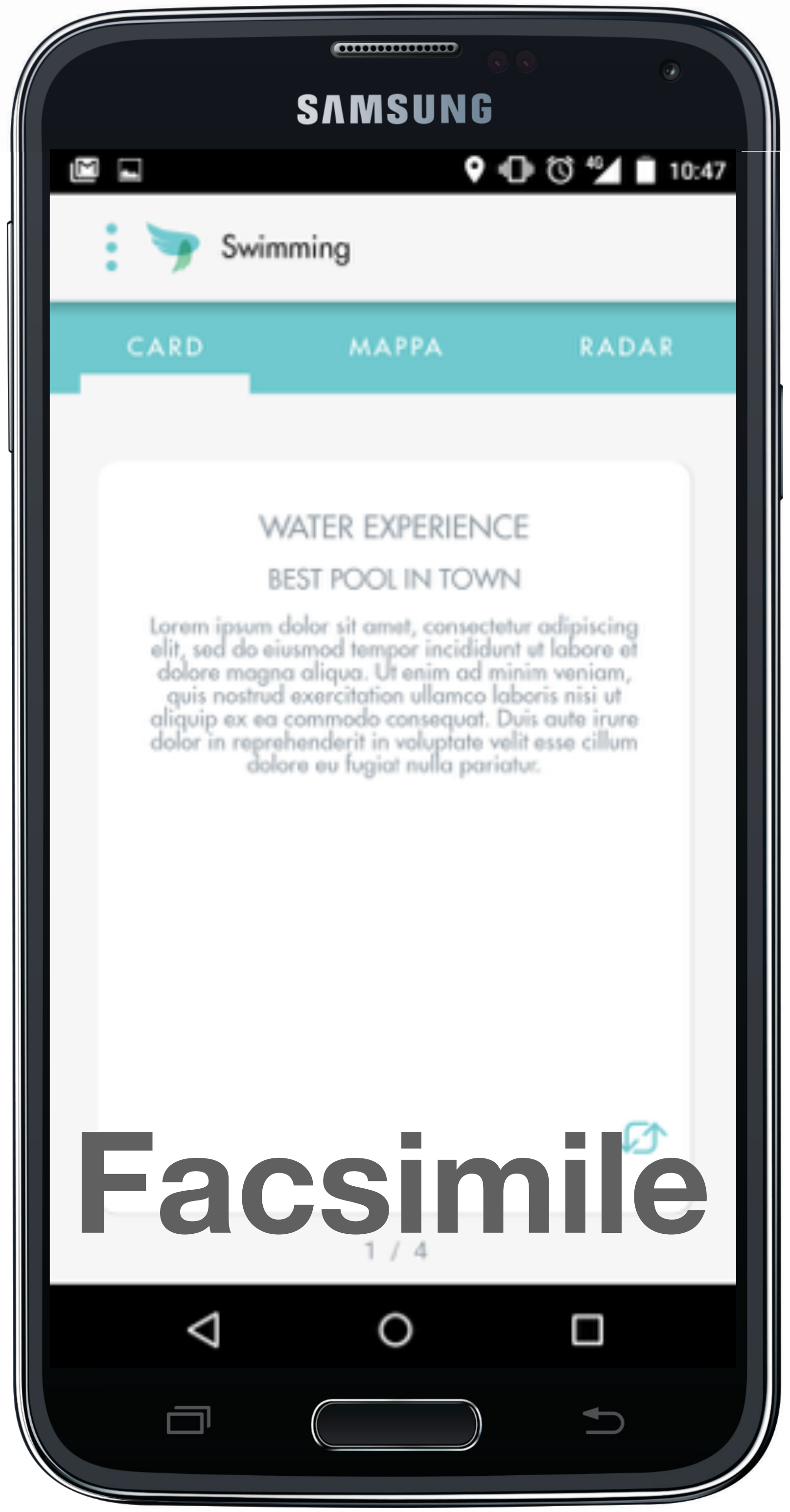

Open and browse the app to complete the following task:

1. Find the closest point of interest among the ones available in the app
2. Find out at what time the Point of interest #1 is open
3. Reckon how many points of interest are available in a 5 kilometres range

Answer here

1. The closest point of interest is: ______________________________
2. The opening hours for the Point of interest #1 is: ______________________________
3. In a 5 kilometres range there are ____________ points of interests

Gartner 2013 CoolVendor
RED HERRING GLOBAL 100 WINNER
Neosperience

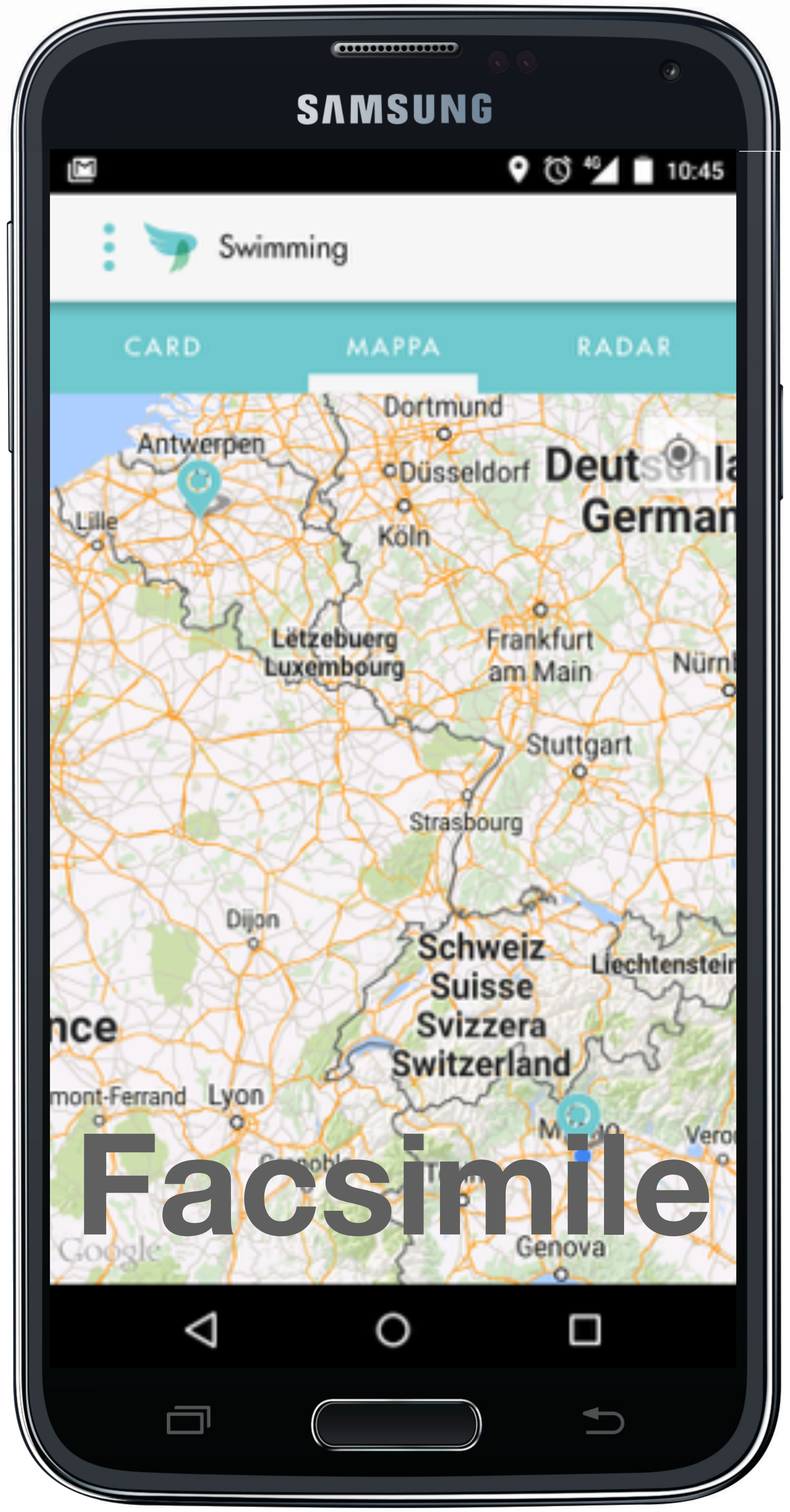

After having used the app answer the following

1. How much is the navigation system is clear?
    1. a lot
    2. enough
    3. not that much
    4. not at all
2. Which navigation view is the most useful to find a place?
    1. card
    2. map
    3. radar
3. Which navigation view is the most fun to use?
    1. card
    2. map
    3. radar
