# Supplementary material for: A Mobile Phone Intervention to Improve Obesity-Related Health Behaviors of Adolescents Across Europe: Iterative Co-Design and Feasibility Study
Source: JMIR Mhealth Uhealth. 2020 Mar 2;8(3):e14118. doi: 10.2196/14118 (PMC7076410; doi:10.2196/14118)
Supplement: Multimedia Appendix 2 [file mhealth_v8i3e14118_app2.docx]

***Multimedia Appendix 2***

# Iterative co-design of an mHealth intervention to improve health behaviors of adolescents across Europe: The PEGASO Fit for Future project

***Focus group discussion guide: 1^st^ iteration***

| **Serious Game** | |
| --- | --- |
| **General presentation of the game** | - What do you think about the idea of this for a game? - What do you think about the “day-night” game idea (opinions about the mood, the mechanics, the icons, etc…)? - Do you like it? If yes, why?, If not, why? - Do you think your friends would like it? Why, which features? |
| **Game mechanics and icons** | - What do you think about these ideas (in particular try to investigate opinions about the mechanics, the icons, etc…)?   - What do you like? Why?   - What don’t you like? Why - Would you play this game? Why? - Would your friends play the game? Why? - During which moments of your day would you like to play this game? Why? - On which device would you like to play this game? Why? - How often would you like to play this game? Why? - Let's say we want you to play for about 15 minutes a day. What do you think would be **most** and **least** important things that might encourage you to do this?   - Being able to develop my character?   - Having a really good story?   - Being able to compete with my friends?   - Being able to share progress and rewards with my friends?   - Knowing I'm improving my lifestyle and health?   - Having fun zapping zombies?   - Something else? |
| **Graphic style** | - What do you think about these different graphic styles? - Which one do you like most? - Why? - Which style do you think is the most appropriate for the game we presented? - Why? - Do you think your friends would agree with you? If not, why not? - Why? |
| **PEGASO Mobile Apps** | |
| **eDiary app** | - What are you looking for when using the app? - What do you think about the interface at that moment? - What is good about the app? – Why? - What is not good about the app? – Why?   Is there something you do not understand or is unclear when using the app? |

***Focus group discussion guide: 2^nd^ iteration***

| **Serious Game** | |
| --- | --- |
| **Talk about how the teenagers found exploring the world in the day, finding the food, and making benefits** | - Who managed to complete the mini-game and make a benefit? - Those of you that didn't, why not? - Those of you that did, how many times did you play the mini-game? - Why did you stop playing it?   - [If needed cue]   - Was it a lack of things to make?   - Was it frustrating?   - Was it difficult to understand? - In combining the foods to make benefits, did you think you learnt anything about what nutrients were in the foods?   - Do you think you learnt that just by playing the game?   - Did you use anything else or ask friends about the nutrients to help? |
| **Talk about the zombies....** | - Did you enjoy fighting them? - What were your favourite parts of combat? - What did you find the most frustrating? - Is there anything you'd like to see in ideas for new attacks or zombie types? - Did you find the game too hard, or too easy? |
| **Talk about things we could add that might make the teenager want to play for longer.** | - What did you think about the leveling up and abilities? Did you look forward to achieving them? - Do you think any abilities should be changed? Which and why? - What did you think about the benefits you made with food? Do you think any should be changed? Which and why? - Do you have any ideas for new abilities or benefits? - Imagine there are already a lot more abilities and benefits in the game, and more types of zombie. What else might be added that you'd like to see - Do you think your friends would be interested in playing too? |

***Focus group discussion guide: 3^rd^ iteration***

| **PEGASO Mobile Apps** | |
| --- | --- |
| **Companion** | Which elements/aspect of the app did you like most? Why?  Which elements/aspects of the app did you like least? Why?  What would you like to change to make the app better?  How did you find adding friends? Are there other functions you would like to have (e.g likes, emoticons)?  How did you find the messages (tips, information, quiz)? |
| **Challenges** | Would you like to have the possibility to create a challenge for your friends? Can you name some examples?  Which kind of challenges did you prefer? (Individual/Collaborative/Competitive) , Why? |
| **Gamification** | Is it clear to you how you can earn points in the Pegaso system?  What is the difference between badges, points and FitCoins?  Would you use FitCoins to buy/have a discount on real products?  For which services would you want a discount (such as gym/swimming/events/sports wear/restaurants)?  How did you find the energy bar (understand how is works, usefulness)? |
| **eDiary** | How did find the graphical presentation and how easy was it to use the app and understand the information?  Do you like the suggestions provided by the eDiary? (food groups/ meals eaten/ recipe suggestions)  How did you find receiving recipes?  Did you use them to prepare a meal? If not, why not?  What else would you like to receive? Missing information in the recipe?  Would you prefer to receive a video receipe?  Do you like getting feedback about whether you are achieving your target goal? How often would you like to be reminded? Would you prefer a notification or a graphical feedback, or both? |

| **Serious Game** | |
| --- | --- |
| **Controlling the character** | Did you understand the controls straight away?  Were they fun to use in running around and fighting the zombies?  Do you think the controls could be improved? How?  Did you find the game too easy or too hard? Why? |
| **Daytime activities in the game** | Did you enjoy the ‘day time’?  Which part did you like most?  Did you like the activity to ‘scavenge for food’?  Did you understand how the research system worked?  Did you manage to upgrade abilities easily? Was it too easy or too hard?  Did you understand the benefits of upgrading abilities?  What was your favourite ability? Why?  What was your least favourite ability? Why? |
| **Game story** | Did you understand what the game was about?  Did you have a good idea of what to do while you were playing?  Were there any times you felt lost or confused? Can you explain?  Did you read the emails? Were they easy to understand? |
| **Game world** | Was it easy to explore the world?  What were your favourite and least favourite parts of the world?  What would you like to see added to the world? |
