# Supplementary material for: A Mobile Phone Intervention to Improve Obesity-Related Health Behaviors of Adolescents Across Europe: Iterative Co-Design and Feasibility Study
Source: JMIR Mhealth Uhealth. 2020 Mar 2;8(3):e14118. doi: 10.2196/14118 (PMC7076410; doi:10.2196/14118)
Supplement: Multimedia Appendix 3 [file mhealth_v8i3e14118_app3.docx]

**Multimedia Appendix 3**

# Iterative co-design of an mHealth intervention to improve health behaviors of adolescents across Europe: The PEGASO Fit for Future project

**The Brief Use of App Questionnaire**

**Companion**

- - - 1. How many messages per day would you like to receive from the final application?
      2. How many reminders per day would you like to receive from the final application?
      3. Which element/aspect of the interaction you liked least?
      4. Which element of the interaction you liked most?

Challenges User Interface:

- - - 1. Do you would like to create a challenge for your friends? Yes / No

If yes, please provide few examples:

- - - 1. Which element/aspect of the interface you liked least?
      2. Which element of the interface you liked most?

**eDiary**

- - - 1. Did reminders help you filling the eDiary? Yes / No
      2. The numbers of food groups is too small or too big?
  - 1-7 (1 too small, 7 too big)
    - 1. Which element/aspect of the interaction you liked least?
      2. Which element of the interaction you liked most?
