# Supplementary material for: A Mobile Phone Intervention to Improve Obesity-Related Health Behaviors of Adolescents Across Europe: Iterative Co-Design and Feasibility Study
Source: JMIR Mhealth Uhealth. 2020 Mar 2;8(3):e14118. doi: 10.2196/14118 (PMC7076410; doi:10.2196/14118)
Supplement: Multimedia Appendix 5 [file mhealth_v8i3e14118_app5.doc]

**Multimedia Appendix 4**

# A smartphone intervention to improve obesity-related health behaviors of adolescents across Europe: An iterative co-design study

**Participants’ quotes**

**Table 1: Feedback about the Companion App**

| **Participant Feedback** | **Example Quotes** |
| --- | --- |
| Liked the overall design | “It looked nice, good design. It fit well with the operating system.” |
| Liked the idea of the Challenge option | “Being able to challenge your friend motivates you I think. Seeing the medals I wouldn’t say is motivating, it’s just, more just pleasing to see that you’ve actually got.” |
| Not understanding the meaning of the Challenge icon; alternative icon design suggested | “This icon is a bit confusing for challenges.” “It [Challenge icon] could be represented by a muscled arm, or a bull’s eye with an arrow.” |
| Liked the idea of having a mascot and selected a favorite design | “The big eyes are nicer, it looks cute.” “I ticked the wee [little] cute one.” |
| Having a customizable avatar | “I think the wee [little] companion person that sort of represented you, was really helpful. It’s something that I can have a bit of fun with it. I was making my own” |
| Not wanting to share data around eating habits | “I would not like to share what I had been eating.”  “I eat a lot of food, so I would not share it.” |
| Some challenges were difficult, and some were easy; suggested to include incremental challenges | “Setting different levels of difficulties that can be increased across time.” |
| Participants suggested to include a leader-board as they would be more motivated to engage in health behaviors when seeing their friends’ performance | “Probably seeing other people’s scores and that they’ve like walked, it would motivate me to do that cause I’m quite competitive.” |
| Did not like to receive too many notifications and/or messages | “Not too frequent, otherwise they just get a bit annoying.” |
| Frustration when receiving notification when it is not possible to respond; suggested to receive notifications in after school hours | “If it would be once, at the end of the day or something. Everyone chills at the end of the day and then get notifications to put in things in the eDiary instead it’s gone off during the day…when you are at school you might be busy.” |
| Needing a tutorial or guidance for use of the app | ”A tutorial would be appreciated.” |

**Table 2.** **Feedback about the Serious Game**

| **Participant Feedback** | **Example Quotes** |
| --- | --- |
| Liked the concept of the game but it was too repetitive with lack of progression | “My main issue with the game is just having a bit of progression and a bit more diversity of activities and what you could do. But other than that, the concept of the game, the idea behind it, it okay. I wouldn’t change that.”  ”It should just change at some point. It is all the same. When you stay live when fighting the zombies and move away from them, you should change environments.” |
| Suggested to provide information about the narrative and purpose of the game | “If you had a story to go with and an end point your knew you had to go to that would be good ‘cause that would make you want to finish the game.” |
| suggested to add a tutorial on what the game is about | “I was kind of wandering around [..] until it got dark and I was attacked by zombies…You needed like a tutorial at the start.”  “Then it didn’t like give you a tutorial on how to do anything. It dumped you straight in the game.” |
| The game world is too easy to explore (suggested to provide further dynamism and complexity; specific design ideas for environments/locations provided) | “I think there should be different things and all of the map just looked like exactly the same…maybe extend the map so it’s in different surroundings, it could be like a city or maybe like a desert or something.” |
| Repeating the same mini game was boring | “It does add variation, but at the end of the day, it was still the same mini game.”  “I just think there wasn’t enough variation between, you know, like, it was the same, daytime, mini games, night time, you know.” |
| Some participants noted they were progressing to higher levels, but they did not notice clear differences between the levels; suggested to add new type of zombies, environments and abilities (specific ideas provided) with each level | “You had ones [zombies] that were slower, and a few ones that were fast. It was kind of, everything a zombie does, some were fast, some were slow. It was nothing really special.” |
| Suggested to add audio features and sounds | “It was also like, really dull, really quiet in the game. There wasn’t any music or sound.” |

**Table 3:** **Feedback about the eDiary App**

| **Participant Feedback** | **Example Quotes** |
| --- | --- |
| Participants liked the app and preferred it over other PEGASO F4F apps | “I didn’t really use any app apart from the e-diary.” If it was on the market and it was like free or even paying…it’s worth paying for.”  “It’s nice and colorful and it looks really good.”  “I think it’s like interesting ‘cause sometimes you’d log in things and then you like wouldn’t realise there’s that much sugar in it, but the sugar would go up quite a bit.” |
| It was difficult for adolescents to understand the servings and food included; suggested to include a tutorial | “Pop-up instruction [are needed] when the app is opened for the first time where it is explained how to enter food using an example meal.” |
| Preference of symbolic food icon over food images | “I don’t like the pictures [of food], use icons instead.” |
| Not understanding the meaning of “equilibrium” | “…equilibrium, diversity…I don’t know what that means.”  “It needs easy to understand words.” |
| Wanting suggestions on how to improve their diet | “In spite of eating cookies…to have some orientation on alternatives.”  “Recommendations were always the same, even when changing the eating habits.” |
| Participants proposed to increase the number of food groups and suggested alternative food groups | “There weren’t enough food groups to choose from.” |
